# Supplementary material for: Integration of photocatalytic and dark-operating catalytic biomimetic transformations through DNA-based constitutional dynamic networks
Source: Nat Commun. 2021 Jul 9;12:4224. doi: 10.1038/s41467-021-24512-y (PMC8270929; doi:10.1038/s41467-021-24512-y)
Supplement: Supplementary file 1 — Supplementary Information [file 41467_2021_24512_MOESM1_ESM.pdf]

Supplementary Information

Integration of photocatalytic and dark-operating catalytic biomimetic transformations through DNA-based constitutional dynamic networks

Chen Wang,[1] Michael P. O'Hagan,[1] Ehud Neumann,[2] Rachel Nechushtai[2] and Itamar Willner[1]*

[1] Institute of Chemistry, The Minerva Center for Complex Bio-hybrid Systems, The Hebrew University of Jerusalem, Jerusalem 91904, Israel. E-mail: itamar.willner@mail.huji.ac.il

[2] Institute of Life Science, The Hebrew University of Jerusalem, Jerusalem 91904, Israel

Contents

Chemicals 3

Instrumentation 3

Experimental Section 4

Synthesis of 1-(3-aminopropyl)-1'-methyl-[4,4'-bipyridine]-1,1'-diium (V^{2+}) 4

Modification of strand A′ with V^{2+} 4

Modification of strand D with LDH 4

Modification of strand D′ with NAD^{+} 5

Preparation of CDNs 5

Formation of Hairpin Structures 6

Intercommunication between two CDNs 6

Photosynthesis measurement 7

MB^{+}→MBH reduction guided by CDNs 7

Mass analysis of alanine generated by CDNs 8

Supplementary Figures 1-38 and Tables 1-15 8

Supplementary References 59

Chemicals

4-(2-hydroxyethyl)piperazine-1-ethanesulfonic acid sodium salt (HEPES), Tris(hydroxymethyl)aminomethane, tris hydrochloride, magnesium chloride, ammonium chloride, protoporphyrin IX (Zn(II)PPIX), 2-mercaptoethanol, L-lactate dehydrogenase from *rabbit muscle* (LDH, 550 U/mg), alanine dehydrogenase, recombinant, expressed in *E. coli* (AlaDH, 21 U/mg), ferredoxin-$NADP^+$ reductase (FNR, 84 U/mL), nicotinamide adenine dinucleotide (NAD^+), nicotinamide adenine dinucleotide phosphate ($NADP^+$), N-(ε-maleimidocaproyloxy) sulfosuccinimide ester (sulfo-EMCS), succinimidyl 3-(2-pyridyldithio)propionate (SPDP), 4-carboxyphenylboronic acid, 1-ethyl-3-(3-dimethylaminopropyl)carbodiimide (EDC), N-hydroxysulfosuccinimide (NHS), lactic acid, methylene blue (MB^+), hydrazine hydrate, glycine, tris(2-carboxyethyl)phosphine (TCEP) and propionic acid were purchased from Sigma-Aldrich. Nucleic acid strands were purchased from Integrated DNA Technologies Inc. (Coralville, IA). Ultrapure water purified by a NANOpure Diamond instrument (Barnstead International, Dubuque, IA, USA).

Instrumentation

Absorption spectra were recorded at 25 °C using a UV-2450 spectrophotometer (Shimadzu), a cuvette of 50 µL volume (made of quartz suprasil, Hellma Analytics) was used in these experiments.

Fluorescence spectra were recorded at 25 °C using a Cary Eclipse Fluorometer (Varian Inc), a cuvette of 100 µL volume (disposable cuvettes, Brand GMBH, Wehrheim, Germany) was used in these experiments. The excitations of FAM, ROX, Cy5 and Cy5.5 were 496, 588, 648 and 685 nm, respectively. The emissions of FAM, ROX, Cy5 and Cy5.5 were 516, 608, 668 and 706 nm, respectively.

Mass analysis was obtained by liquid chromatography Mass spectroscopy LC(UV)MS using Agilent 6520 QTOF analyzer. Mass data was collected using Mass Hunter Workstation software 6.00. The data was analyzed using Mass Hunter Quantitative Analysis B.06.00. In negative mode to probe pyruvic acid, the mobile phase is 50% methanol and 50% water, and propionic acid was used as an internal standard. In positive mode, the mobile phase is 0.01% aqueous formic acid : H_2O = 1:1 and glycine was used as an internal standard.

A Xe lamp with a 399 nm cut off filter was used to irradiate the sample. The absorbance spectra

were measured every 5 min upon irradiation. NMR was performed by using a Bruker Ultrashield Plus 500 MHz spectrometer.

The base-pairing probabilities were analyzed using an online platform, NUPACK web application (http://www.nupack.org/).

Experimental Section

Synthesis of 1-(3-aminopropyl)-1'-methyl-[4,4'-bipyridine]-1,1'-diium (V^{2+})

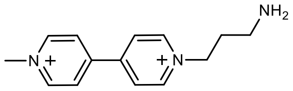

V^{2+} was synthesized according to the previous literature.[1] ^{1}H NMR (500 MHz, D_2O): δ 2.46 ppm (m, 2H), 3.16 ppm (t, 2H), 4.47 ppm (s, 3H), 4.82 ppm (t, 2H), 8.49 ppm (d, 2H), 8.55 ppm (d, 2H), 9.03 ppm (d, 2H), 9.14 ppm (d, 2H) (Supplementary Figure 1).

Modification of strand A′ with V^{2+}

Before conjugation with V^{2+}, strand A′ was treated with TCEP for 2 hours. 60 μL of 0.05 M V^{2+} (10 eq) and 30 μL of 0.01 M sulfo-EMCS (1 eq) were mixed in PBS buffer (20 mM, pH = 7.24) and incubated at room temperature for 1 hour. Then 30 μL of 1 mM strand A′ (0.1 eq) was added and incubated for another 2 hours. Excess reactants were removed using Amicon 10 kD cutoff filters. The synthesis route was shown in Supplementary Figure 2a. The characterization of the modified strand was shown in Supplementary Figure 35, 36 and Supplementary Table 10.

Modification of strand D with LDH

10 μM of LDH and 1.2 mM SPDP in HEPES buffer (10 mM, pH = 8) were incubated for 1 hour. Excess SPDP was removed using Amicon 30 kD cutoff filters. Before modification of strand D with LDH, strand D was treated with TCEP (100-fold excess) for 2 hours and washed by using Amicon 3 kD cutoff filters. Next, SPDP-modified LDH was conjugated to strand D (8-fold excess) through a disulfide bond exchange of the activated pyridyldithiol group (see synthetic scheme in Supplementary Figure 2b). The reaction was performed in HEPES buffer (10 mM, pH = 8) for 2 hours. The SPDP coupling efficiency was evaluated by monitoring the increase in absorbance at 343 nm due to the release of pyridine-2-thione (extinction coefficient: 8,080 M^{-1} cm^{-1}), Supplementary Figure 8a-b.

Excess DNA was removed using Amicon 30 kD cutoff filters. The enzymatic activity of DNA-modified LDH was ~ 75% of the activity of the native enzyme (Supplementary Figure 8c). The DNA labeling ratio of the purified enzyme-DNA conjugates was estimated by measuring the absorbance at 260 and 280 nm (Supplementary Figure 37 and Supplementary Table 11) and mass analysis was shown in Supplementary Figure 38.

Modification of strand D′ with NAD$^+$

The preparation of D′-NAD$^+$ was followed our previous paper and characterized by mass spectrum.[2] The detailed preparation of D′-NAD$^+$ (see synthetic scheme in Supplementary Fig. 2c) was in the following. 4-carboxyphenylboronic acid (1.8 μL, 50 mM) reacted with EDC (2.3 μL, 10 mg/mL) in 200 μL of MES buffer (10 mM, pH = 5.5) for 5 min, subsequently NHS (3.5 μL, 10 mg/mL) was added and reacted for 10 min. Then, D′ (2.3 μL, 4.4 mM) and NAD$^+$ (3 μL, 5 mM) were added, stirring for 2 hours and kept at 4 °C overnight. Excess reactants were washed away with Amicon 10 kD cutoff filters.

Preparation of CDNs

A sample of 1 mL of CDN (each component 2 μM) was taken as an example to explain the procedure of the preparation of CDNs:

CDN X, including the constituents AA′, BB′, AB′, BA′, was prepared as follows: A (20 μL, 100 μM), A′ (20 μL, 100 μM), B (20 μL, 100 μM), B′ (20 μL, 100 μM) and PPIX (2 μL, 1 mM) were mixed in Tris buffer (10 mM, pH = 7.29) that includes 20 mM $MgCl_2$ and 100 mM K^+. The mixture was annealed at 37 °C, cooled down to 25 °C at a rate of 0.33 °C/min and equilibrated at 25 °C for 12 h.

CDN Y, including the constituents LDH/NAD$^+$-DD′, LDH-DC′, CD′-NAD$^+$, CC′ was prepared as follows: LDH-D (20 μL, 100 μM), D′-NAD$^+$ (20 μL, 100 μM), C (20 μL, 100 μM), C′ (20 μL, 100 μM) were mixed in Tris buffer (10 mM, pH = 7.29) that includes 20 mM $MgCl_2$ and 100 mM K^+. The mixture was annealed at 37 °C for 1 hour, cooled down to 25 °C at a rate of 0.33 °C/min, and equilibrated at 25 °C for 12 h.

For the triggered transition of CDN X, triggers T_1, T_1' or T_2, T_2' are 1.67-fold excess than each

component of CDN. After adding triggers into initial CDN, the final concentration of each component of CDN was 1 µM and the final concentration of trigger was 1.67 µM. The solution was incubated at 28°C overnight to equilibrate. For the triggered transition of CDN Y, triggers T_3, T_3' or T_4, T_4' are 2.5-fold excess than each component of CDN. After adding triggers into initial CDN, the final concentration of each component of CDN was 1 µM and the final concentration of trigger was 2.5 µM. The solution was incubated at 28°C overnight to equilibrate. After equilibration, the equilibrated CDN (each component 1 µM) was treated with one substrate (5 µM) (sub 1 for AA′, sub 2 for BB′, sub 3 for BA′, sub 4 for AB′, sub 5 for DC′, sub 6 for CD′, sub 7 for CC′ and sub 8 for DD′). The time-dependent fluorescence changes generated by the cleavage of the different substrates by DNAzyme reporter units were measured. By following the rate of formation of the fluorophore-labeled fragment and using appropriate calibration curves of the intact constituent (Supplementary Figs. 4-5 and 9-10), the quantitative evaluation of the concentrations of constituents is achieved.

Formation of Hairpin Structures

Hairpins H_a, H_d or H_n in Tris buffer (10 mM, pH = 7.29) that includes 20 mM $MgCl_2$ and 100 mM K^+ was annealed at 95 °C for 5 min, cooled down quickly in ice and equilibrated at 25 °C for 2 hours.

Intercommunication between two CDNs

The mixture of two CDNs (1 mL, each component 2 µM) was annealed at 37 °C for 1 hour, cooled down to 25 °C at a rate of 0.33 °C/min, and allowed to equilibrate at 25 °C for 12 h, then subjected to hairpin H_a (33.4 µL, 100 µM) and hairpin H_d (50 µL, 100 µM) in 916.6 µL of Tris buffer (10 mM, pH = 7.29) that includes 20 mM $MgCl_2$ and 100 mM K^+. The final concentration of each component of CDNs was 1 µM. The solution was equilibrated at 28 °C at different time intervals, the sample was withdrawn from the mixture and treated with one substrate (5 µM). The time-dependent fluorescence changes originated by the cleavage of the different substrates by the DNAzyme reporters were measured. Using the appropriate calibration curves of the intact constituent, the contents of the constituents in the different CDNs could be quantitatively evaluated.

Photosynthesis measurement

For probing the generation of $V^{+\bullet}$ radicals by equilibrated CDN X, X_a or X_b, the reaction was performed using equilibrated CDN (each component 5 µM) in 10 mM Tris buffer (pH 7.29, 100 mM K^+, and 20 mM Mg^{2+}) that includes 2-mercaptoethanol (20 mM). All samples were deaerated by bubbling argon for 30 min. A Xe lamp with a 399 nm cut off filter (20 mW) was used to irradiate the sample. The absorbance spectra were measured every 5 min upon irradiation.

For probing the generation of NADPH by CDN X, X_a or X_b, the reaction was performed using equilibrated CDN (each component 5 µM) in 10 mM Tris buffer (pH 7.29, 100 mM K^+, and 20 mM Mg^{2+}) that includes 2-mercaptoethanol (20 mM), $NADP^+$ (0.5 mM) and FNR (8.4 U/mL). All samples were deaerated by bubbling argon for 30 min. A Xe lamp with a 399 nm cut off filter (20 mW) was used to irradiate the sample. The absorbance spectra were measured every 5 min upon irradiation.

For generation of NADPH by two intercommunicated CDNs X and Y, after different time intervals of intercommunication, the biocatalyzed-reduction of NADPH was performed using CDN mixture (each component 5 µM) in 10 mM Tris buffer (pH 7.29, 100 mM K^+, and 20 mM Mg^{2+}) that includes 2-mercaptoethanol (20 mM), $NADP^+$ (2 mM) and FNR (8.4 U/mL). All samples were deaerated by bubbling argon for 30 min. A Xe lamp with a 399 nm cut off filter (20 mW) was used to irradiate the sample. The absorbance spectra were measured every 5 min upon irradiation. (Note: the enzyme LDH included in CDN Y does not react with $NADP^+$ cofactor, presented in CDN X).

$MB^+ \rightarrow$MBH reduction guided by CDNs

For $MB^+ \rightarrow$ MBH reduction guided by equilibrated CDN Y/CDN Y_a/CDN Y_b, the reaction was performed using equilibrated CDN (each component 0.125 µM) in 10 mM Tris buffer (pH 7.29, 100 mM K^+, and 20 mM Mg^{2+}) that includes lactic acid (0.625 mM), hydrazine (0.625 mM), and MB^+ (62.5 µM). The absorbance changes at 630 nm were measured. For probing the generated pyruvic acid, mass analysis was performed in negative mode and propionic acid was used as an internal standard. The amount of generated pyruvic acid was calculated based on the integrated area ratio between m/z = 87 (pyruvic acid) and m/z = 73 (propionic acid).

For $MB^+ \rightarrow MBH$ reduction driven by two intercommunicated CDNs X and Y, after different time intervals of intercommunication, the biocatalyzed-reduction of MBH was performed using CDN mixture (each component 0.2 µM) in 10 mM Tris buffer (pH 7.29, 100 mM K^+, and 20 mM Mg^{2+}) that includes lactic acid (0.455 mM), hydrazine (0.455 mM), and MB^+ (90 µM). The absorbance changes at 630 nm were measured.

Mass analysis of alanine generated by CDNs

For mass analysis, glycine (positive mode) or propionic acid (negative mode) was added to each sample as the internal standard. The amount of L-alanine generated by different CDN systems was evaluated based on the integrated area ratio between mass peak of L-alanine and mass peak of glycine or mass peak of propionic acid. By using appropriate calibration curves, the concentration of product L-alanine was calculated. The integrated area values of different mass peaks were summarized in Supplementary Tables 3-9.

For probing alanine generated by CDN Y, the reaction was performed using equilibrated CDN (each component 1 µM) in 10 mM Tris buffer (pH 7.29, 100 mM K^+, and 20 mM Mg^{2+}) that includes lactic acid (1 mM), hydrazine (1 mM), NH_4Cl (1 mM), glycine (0.78 mM) and alanine dehydrogenase (78 U/mL).

For probing alanine generated by two intercommunicated CDNs X and Y, after different time intervals of intercommunication, the amination of pyruvate was performed using equilibrated CDN (each component 0.2 µM) in 10 mM Tris buffer (pH 7.29, 100 mM K^+, and 20 mM Mg^{2+}) that includes lactic acid (0.2 mM), hydrazine (0.2 mM), NH_4Cl (0.2 mM), glycine (0.4 mM) and alanine dehydrogenase (15.625 U/mL).

Supplementary Figures 1-38 and Tables 1-15

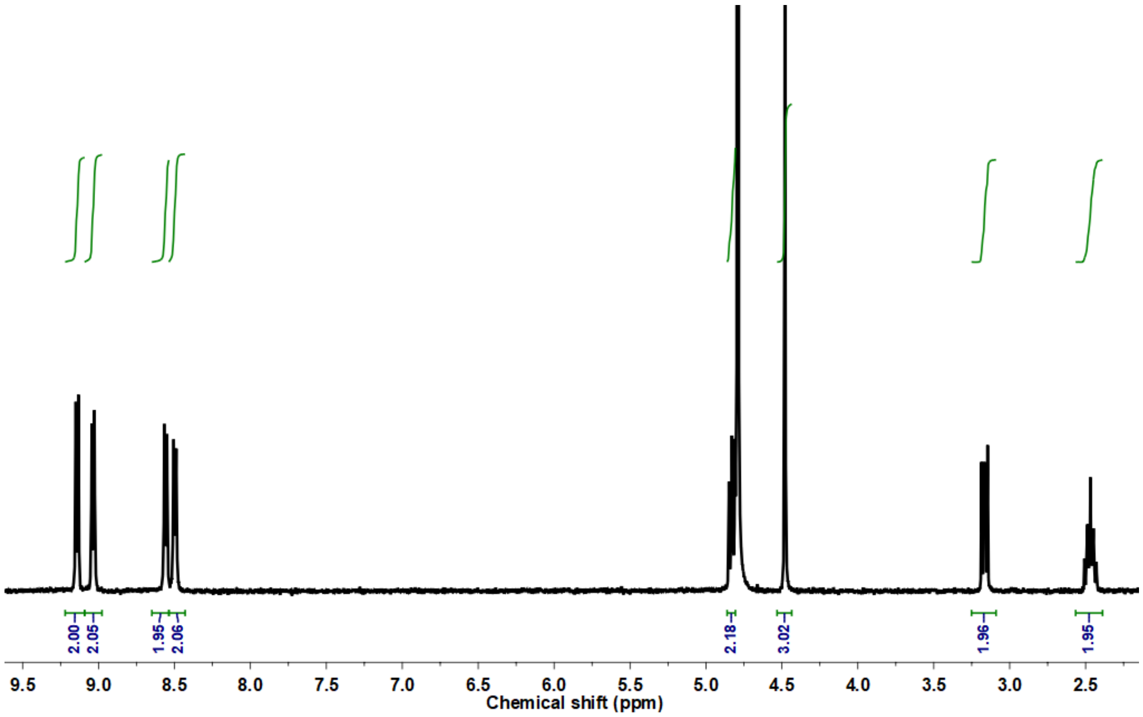

Supplementary Figure 1. ^{1}H NMR spectrum of V^{2+} in D_2O.

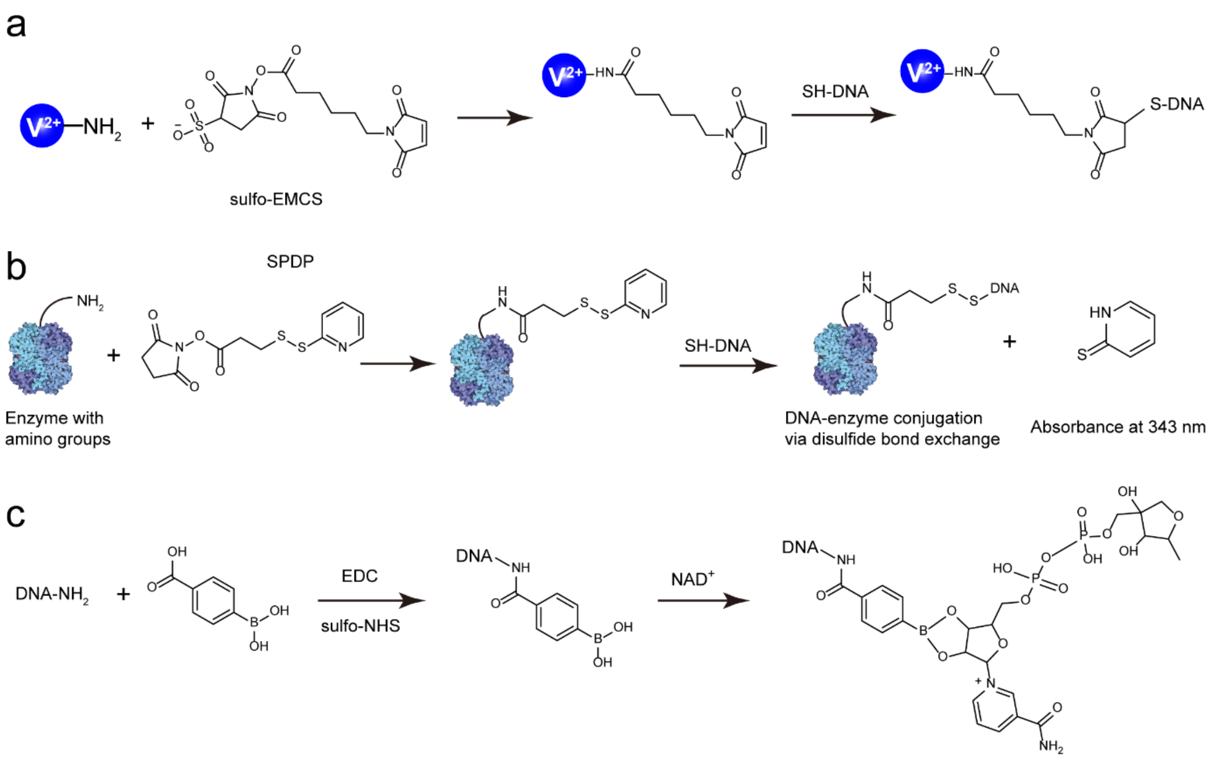

Supplementary Figure 2. **Modification of different DNA strands.** (a) Synthesis route of LDH-DNA conjugates. (b) Synthesis route of V^{2+}-DNA conjugates. (c) Synthesis route of NAD^{+}-DNA conjugates.

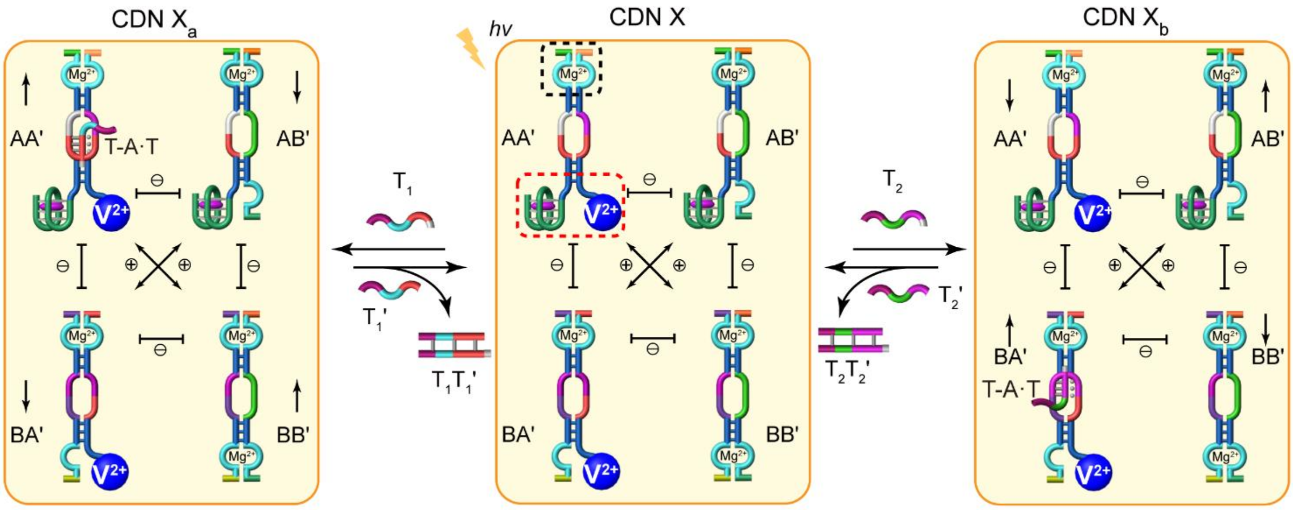

Supplementary Figure 3. **Triggered reversible reconfiguration of the CDN X in different states.** Triggered reversible reconfiguration of the parent CDN X into equilibrated CDN X_a or CDN X_b, using triggers T_1 or T_2, and T_1' or T_2' as counter triggers, respectively. Zn-PPIX/GQ/MV^{2+} in constituent AA′ activates the photosynthetic process.

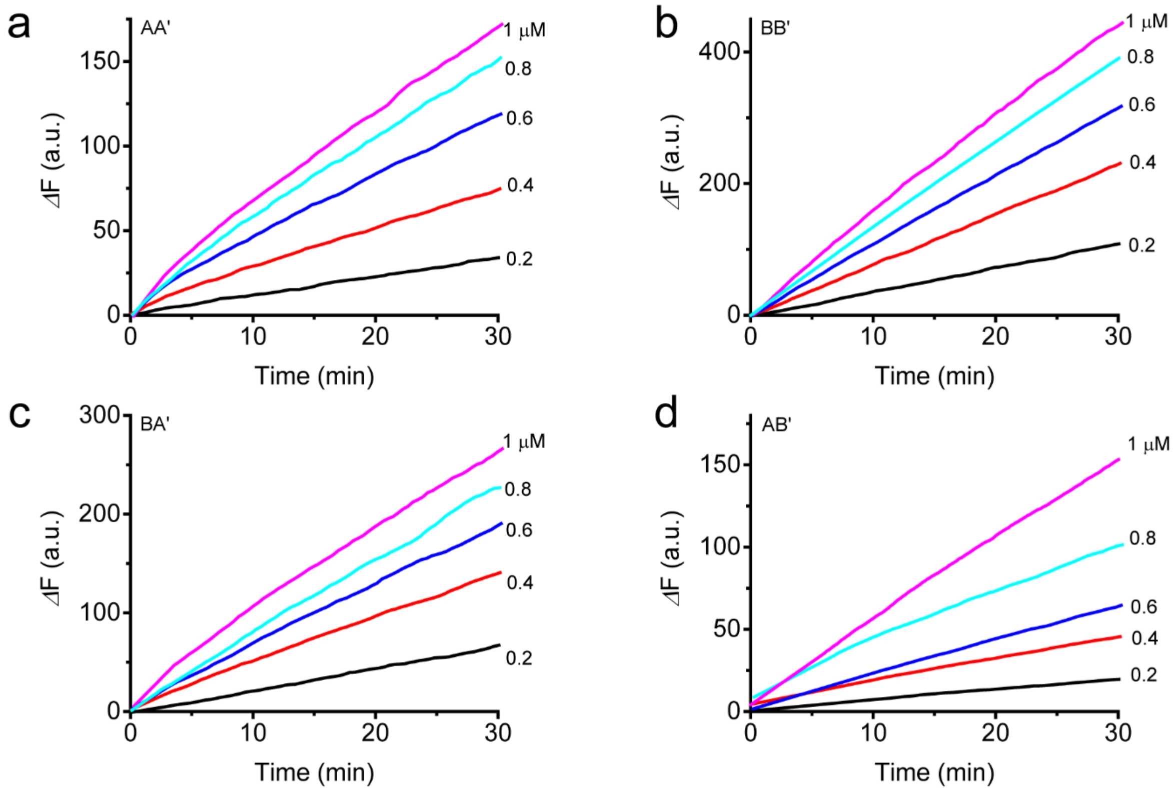

Supplementary Figure 4. **Time-dependent fluorescence changes generated upon cleavage of the respective substrates by variable concentrations of the different DNAzyme reporter units associated with the individual constituents of the CDN X.** For all systems, the concentrations of the DNAzyme reporter units correspond to: 0.20 µM, 0.40 µM, 0.60 µM, 0.80 µM and 1.00 µM. It should be noted that the intact structures AA′-T_1 and BA′-T_2 reveal catalytic activities, similar to those shown for AA′ and BA′, respectively.

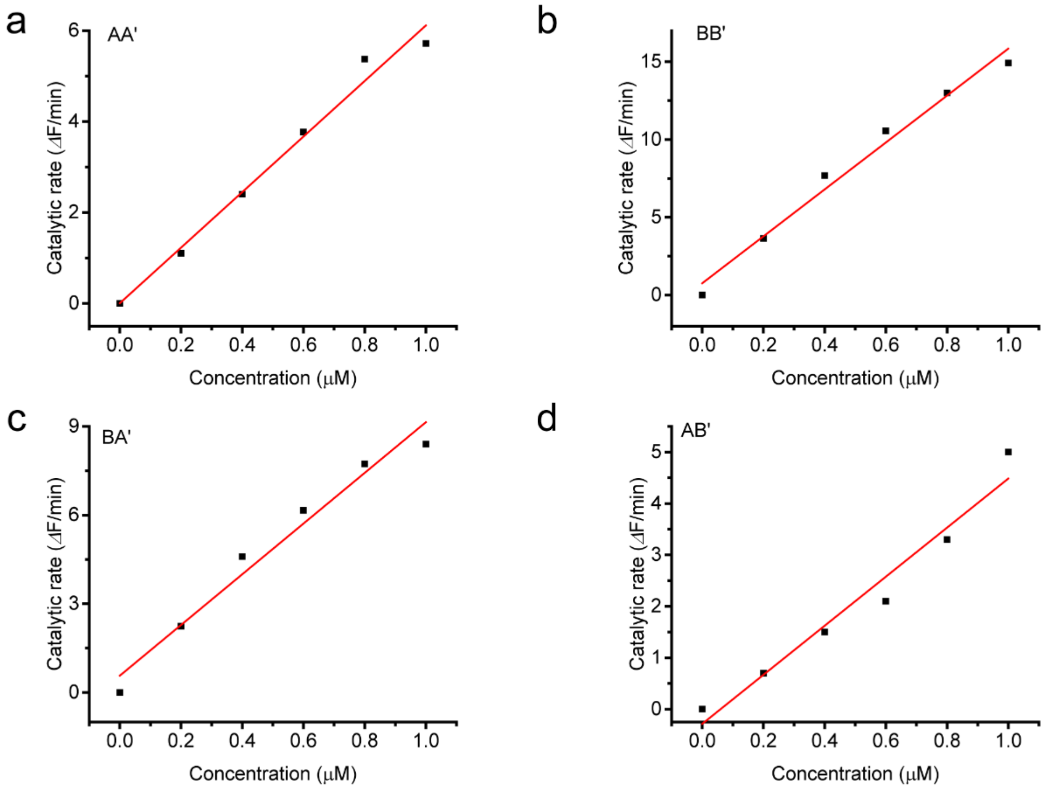

Supplementary Figure 5. Calibration curves corresponding to the rates of the catalytic activities of the different constituents of the CDN X. Calibration curves are derived from the data shown in Supplementary Figure 4.

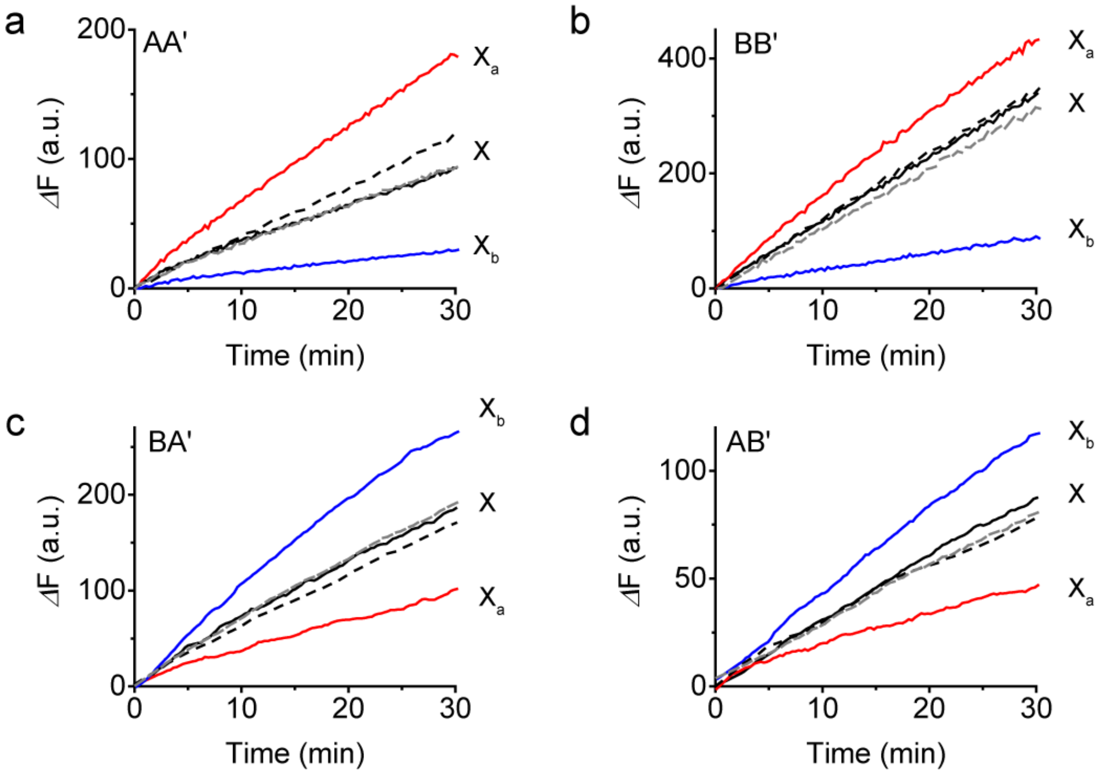

Supplementary Figure 6. Time-dependent fluorescence changes generated from the cleavage of the respective fluorophore/quencher-modified substrates by the DNAzyme reporter units associated with the different constituents in CDN X. (a-d) The time-dependent fluorescence changes of four constituents included in CDN X before (black line) and after (red line) subjecting the CDN X to the trigger T_1 and back to initial state CDN X (dashed black line) upon subjecting to T_1'. The time-dependent fluorescence changes of four constituents included in CDN X after subjecting the CDN X to the trigger T_2 (blue line) and back to initial state CDN X (dashed grey line) upon subjecting to T_2'.

Supplementary Table 1. **Concentrations of the constituents associated with CDN X, CDN X_a and CDN X_b.** Concentration was determined by the DNAzyme reporter units.

System	Concentration (μM)			
	AA′	AB′	BA′	BB′
CDN X	0.49±0.013	0.60±0.015	0.61±0.03	0.62±0.01
CDN X_a	0.92±0.03	0.30±0.005	0.32±0.015	0.89±0.013
CDN X_b	0.18±0.02	0.85±0.03	0.92±0.025	0.13±0.014

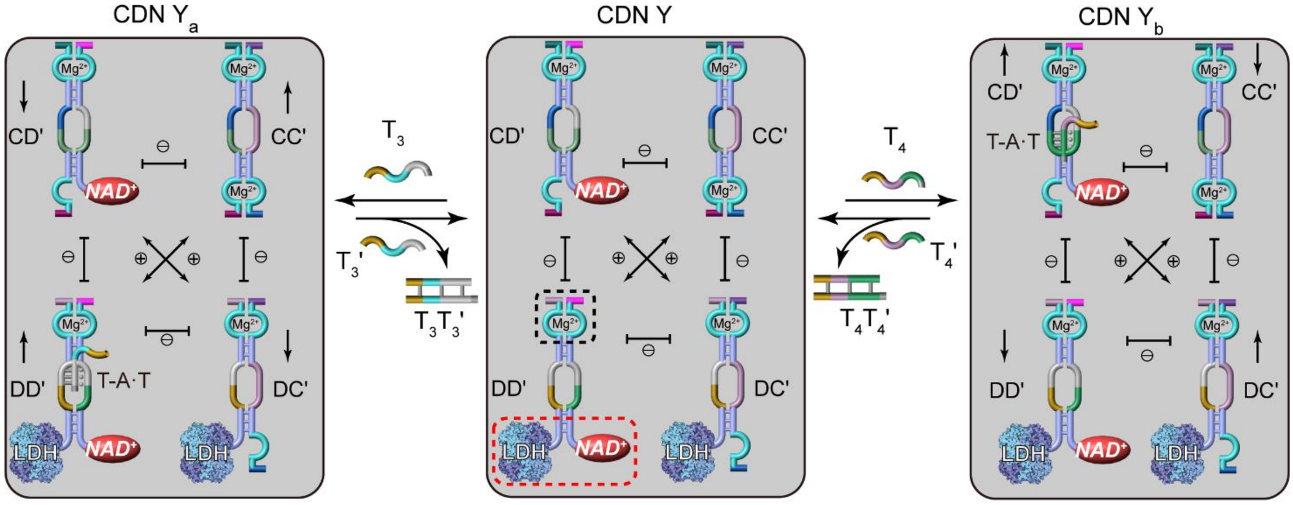

Supplementary Figure 7. **Triggered reversible reconfiguration of the CDN Y in different states.** Triggered reversible reconfiguration of the parent CDN Y into equilibrated CDN Y_a or CDN Y_b, using triggers T_3 or T_4, and T_3' or T_4' as counter triggers, respectively. LDH/NAD$^+$ in constituent DD′ activates the dynamic metabolic process.

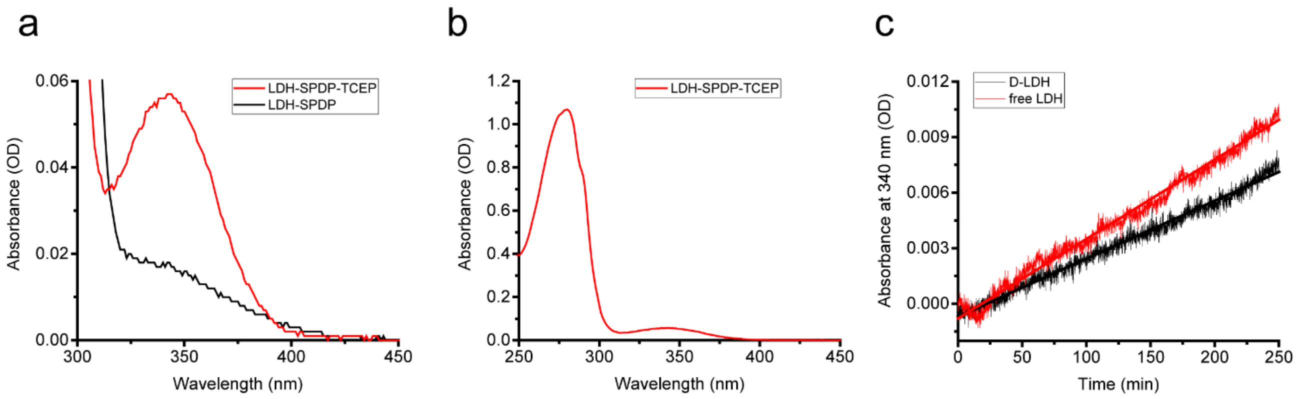

Supplementary Figure 8. **Characterization of LDH-modified strand D**. (a-b) Absorbance spectra of LDH-SPDP before and after the treatment of TCEP: ΔA343 before and after the treatment of TCEP is ~ 0.034 (extinction coefficient of pyridine-2-thione: 8,080 M^{-1} cm^{-1}), corresponding to 4.21 µM SPDP coupled with 5.6 µM LDH (extinction coefficient of LDH at 280 nm is 186,502 M^{-1} cm^{-1}). The ratio of LDH: SPDP is 1.33. TCEP is used to calculate the coupling efficiency between LDH and SPDP due to the release of pyridine-2-thione. (c) The enzyme activity of strand D-modified lactate dehydrogenase (D-LDH) as compared to the native LDH by probing the reduction of NAD^+, in the presence of lactic acid (0.14 M), hydrazine (0.14 M), NAD^+ (55 µM) under the identical concentration of LDH (0.2 µM).

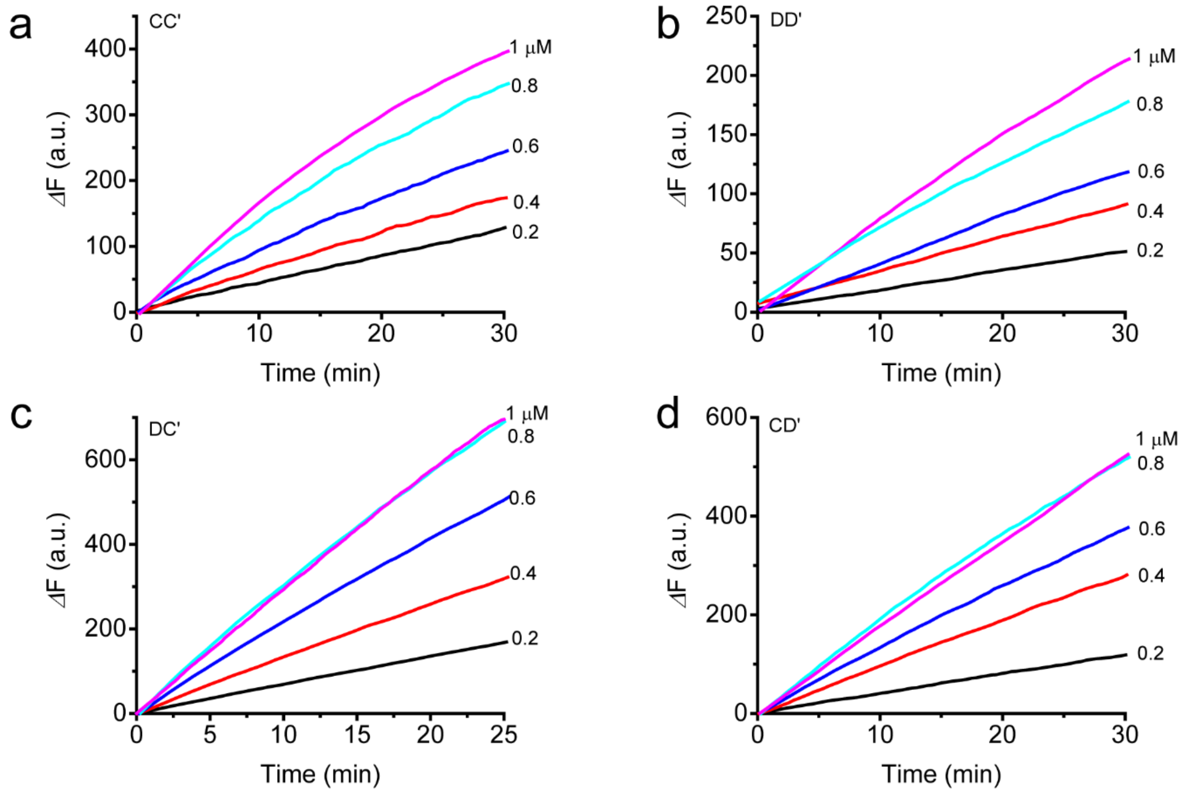

Supplementary Figure 9. **Time-dependent fluorescence changes generated upon cleavage of the respective substrates by variable concentrations of the different DNAzyme reporter units associated with the individual constituents of the CDN Y.** For all systems, the concentrations of the DNAzyme reporter units correspond to: 0.20 μM, 0.40 μM, 0.60 μM, 0.80 μM and 1.00 μM. It should be noted that the intact structures DD′-T_3 and CD′-T_4 reveal catalytic activities, similar to those shown for DD′ and CD′, respectively.

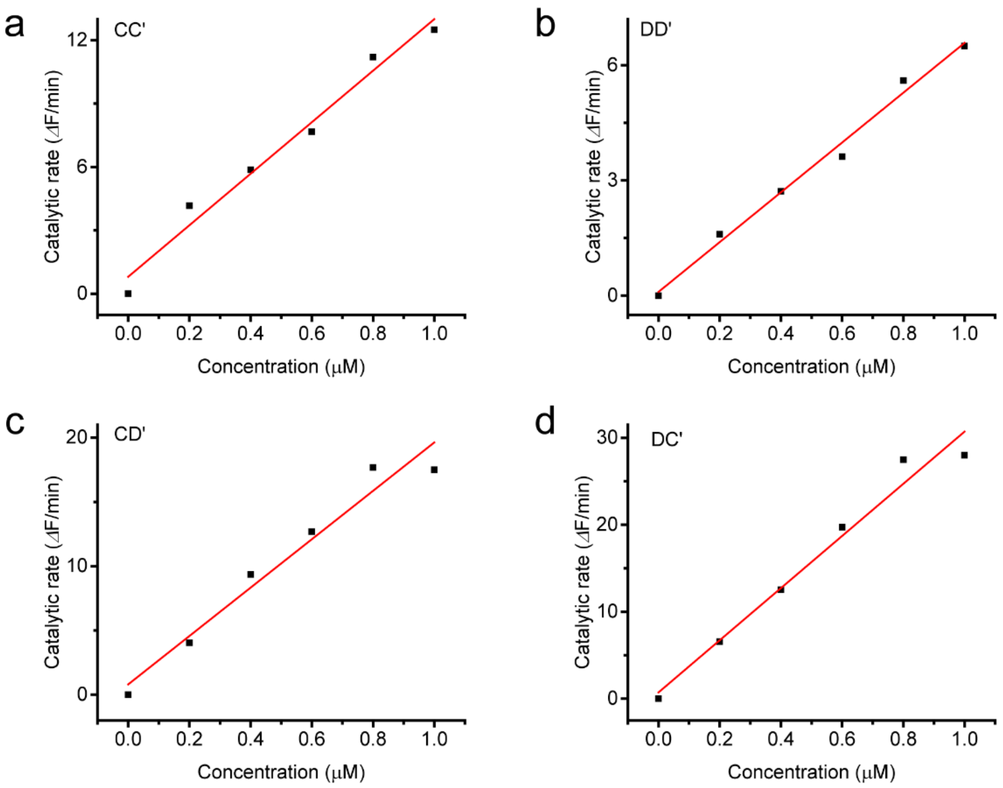

Supplementary Figure 10. Calibration curves corresponding to the rates of the catalytic activities of the different constituents of the CDN Y. Calibration curves are derived from the data shown in Supplementary Figure 9.

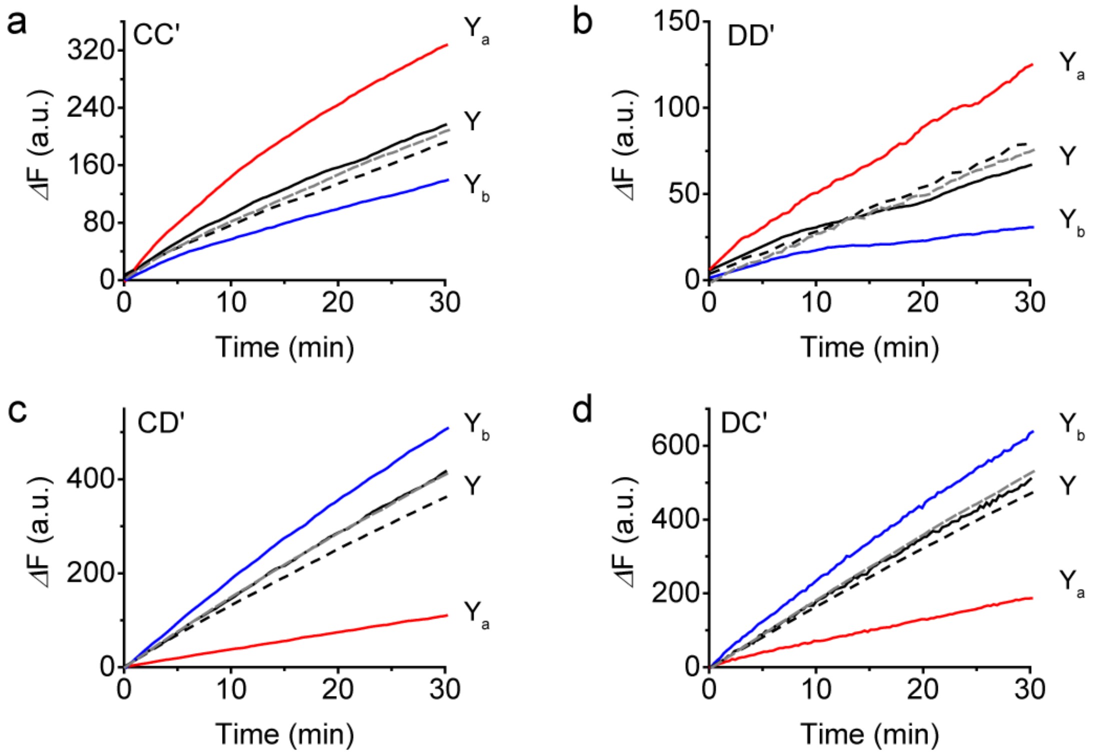

Supplementary Figure 11. Time-dependent fluorescence changes generated from the cleavage of the respective fluorophore/quencher-modified substrates by the DNAzyme reporter units associated with the different constituents in CDN X. (a-d) The time-dependent fluorescence changes of four constituents included in CDN Y before (black line) and after (red line) subjecting the CDN Y to the trigger T_3 and back to initial state CDN Y (dashed black line) upon subjecting to T_3'. The time-dependent fluorescence changes of four constituents included in CDN Y after subjecting the CDN Y to the trigger T_4 (blue line) and back to initial state CDN Y (dashed grey line) upon subjecting to T_4'.

Supplementary Table 2. **Concentrations of the constituents associated with CDN Y, CDN Y_a and CDN Y_b.** Concentration was determined by the DNAzyme reporter units.

System	Concentration (μM)			
	CC′	CD′	DC′	DD′
CDN Y	0.53±0.018	0.68±0.023	0.52±0.02	0.33±0.028
CDN Y_a	0.86±0.04	0.193±0.025	0.20±0.01	0.69±0.06
CDN Y_b	0.28±0.03	0.88±0.025	0.72±0.03	0.16±0.02

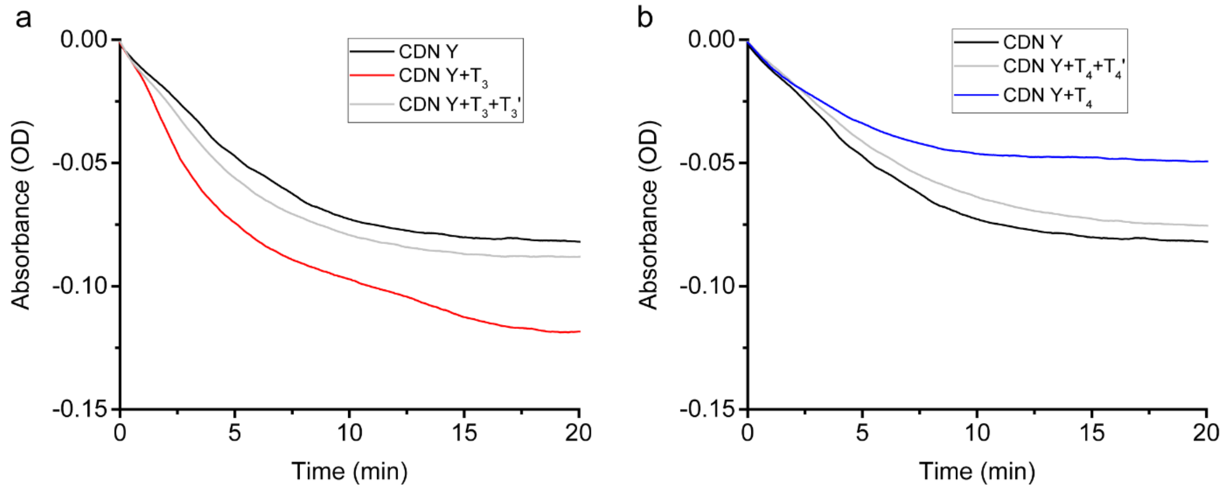

Supplementary Figure 12. **Time-dependent absorbance changes generated by LDH/NAD$^+$/MB$^+$ cascade in different CDN states.** (a) Time-dependent absorbance changes generated by LDH/NAD$^+$/MB$^+$ cascade in different states of CDN Y→ CDN Y_a → CDN Y. T_3-triggered reconfiguration of CDN Y to CDN Y_a enhanced the reduction of MB$^+$ (red curve) and the reverse treatment of CDN Y_a with T_3' regenerated initial CDN Y and regenerated the catalytic reduction of MB$^+$ (grey curve). (b) Time-dependent absorbance changes generated by LDH/NAD$^+$/MB$^+$ cascade in different states of CDN Y→ CDN Y_b → CDN Y. T_4-triggered reconfiguration of CDN Y to CDN Y_b decreased the reduction of MB$^+$ (blue curve) and the reverse treatment of CDN Y_b with T_4' regenerated initial CDN Y and regenerated the catalytic reduction of MB$^+$ (grey curve).

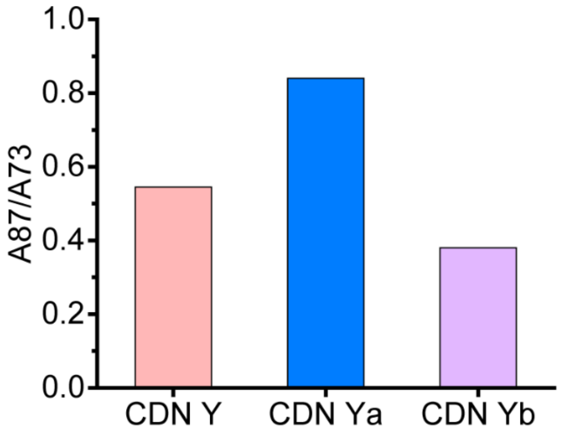

Supplementary Figure 13. **The amount of pyruvic acid generated by LDH/NAD^+/MB^+ cascade in different states of CDN Y.** A87 is the integrated area of mass peak at m/z = 87 (pyruvic acid), A73 is the integrated area of mass peak at m/z = 73 (propionic acid acts as an internal standard for mass analysis of pyruvic acid under negative mode).

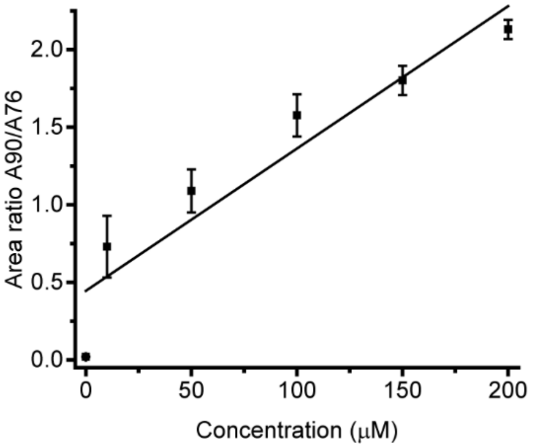

Supplementary Figure 14. **Calibration curve of the relations between the integrated area ratio A90/A76 (m/z = 90 (L-alanine) and m/z = 76 (glycine)) and different concentrations of L-alanine.** The calibration curve is used for the calculation of the concentration of L-alanine generated by CDN Y. Error bars, mean ± s.d. based on three independent experiments.

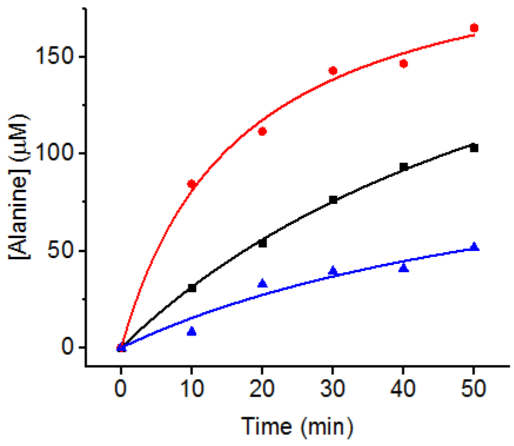

Supplementary Figure 15. **Duplicate results of the experiments shown in Figure 2d.** Time-dependent formation of L-alanine by the metabolic path (2) using the different CDNs Y, Y_a and Y_b.

Supplementary Table 3. **Mass analysis of L-alanine generated by CDN Y.** A90 is the integrated area of mass peak at m/z = 90 (L-alanine), A76 is the integrated area of mass peak at m/z = 76 (glycine as an internal standard, positive mode).

Time (min)	CDN Y		
	A90	A76	A90/A76
10	106807	153121	0.6975
	124731	170689	0.73
20	173088	200654	0.86
	157040	166957	0.94
30	119048	98396	1.2
	166894	134737	1.15
40	179999	146543	1.23
	104325	79889	1.306
50	100232	71166	1.408
	133964	96813	1.394

Supplementary Table 4. Mass analysis of L-alanine generated by CDN Y_a. A90 is the integrated area of mass peak at m/z = 90 (L-alanine), A76 is the integrated area of mass peak at m/z = 76 (glycine as an internal standard, positive mode).

Time (min)	CDN Y_a		
	A90	A76	A90/A76
10	90539	72246	1.25
	77293	63265	1.22
20	116395	80037	1.45
	98264	66587	1.47
30	288601	164234	1.757
	144973	85614	1.7
40	234941	130000	1.8
	108516	60601	1.79
50	139394	71122	1.96
	152705	78765	1.938

Supplementary Table 5. **Mass analysis of L-alanine generated by CDN Y_b.** A90 is the integrated area of mass peak at m/z = 90 (L-alanine), A76 is the integrated area of mass peak at m/z = 76 (glycine as an internal standard, positive mode).

Time (min)	CDN Y_b		
	A90	A76	A90/A76
10	73170	126921	0.576
	7036	13631	0.52
20	201905	264144	0.76
	98549	131994	0.7466
30	116481	148232	0.785
	145565	180510	0.806
40	107323	133590	0.804
	80409	97903	0.82
50	228722	245228	0.93
	116780	127306	0.92

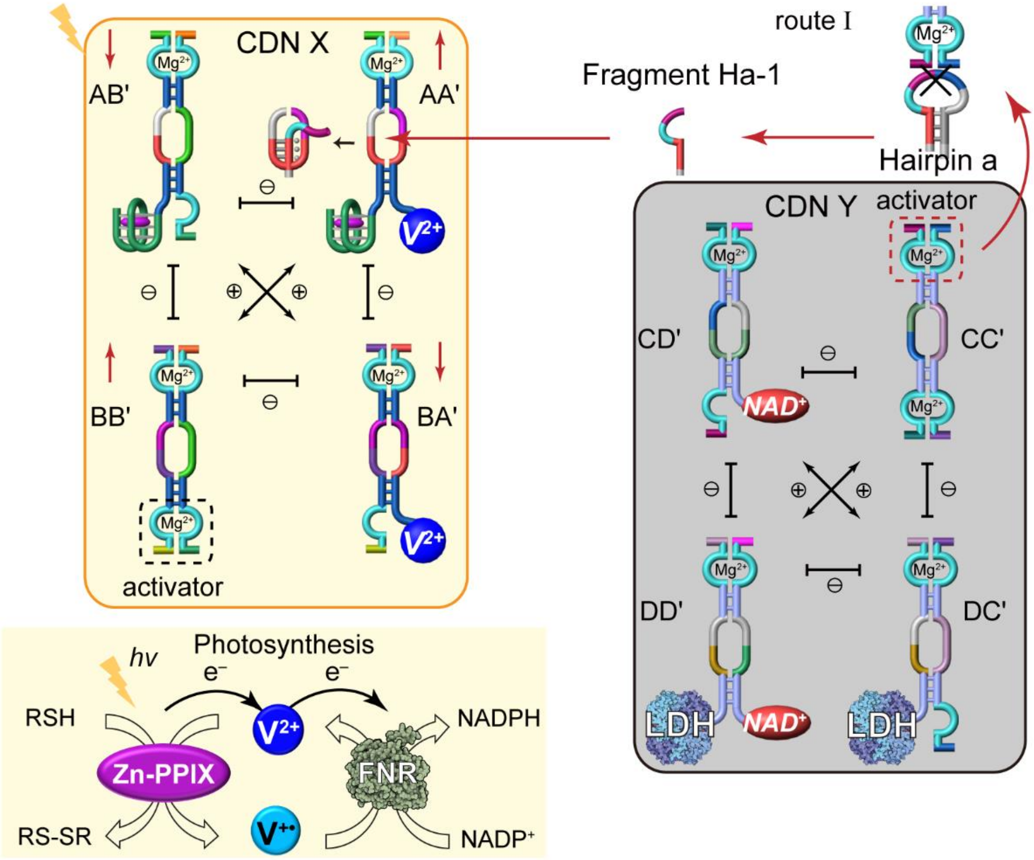

Supplementary Figure 16. **Schematic composition of intercommunicated control between two CDNs using hairpin a (H_a) as a trigger and control over the Zn-PPIX/GQ/MV^{2+} photosynthetic process.** Trigger H_a acts as a substrate for the DNAzyme activator associated with CC′ of CDN Y to yield the fragment strand $H_{a\text{-}1}$ that interacts with AA′, resulting in the up-regulation of AA′ and enhanced activity of photosynthesis.

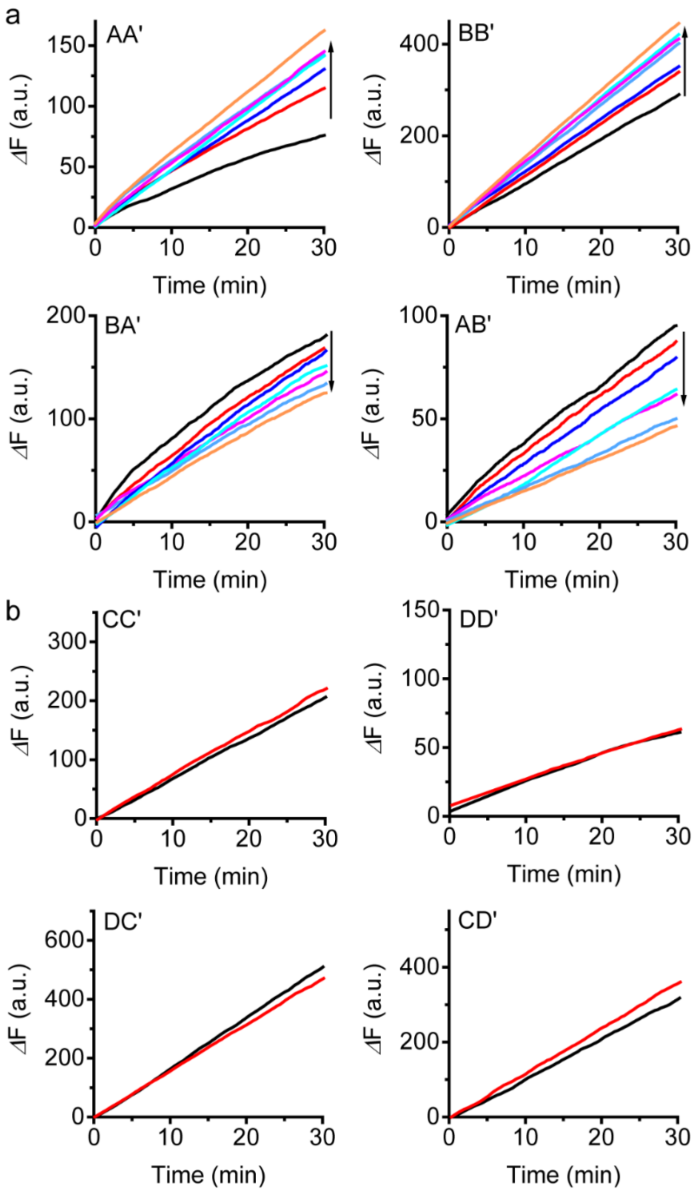

Supplementary Figure 17. **Time-dependent fluorescence changes generated from the cleavage of the respective fluorophore/quencher-modified substrates by the DNAzyme reporter units in the coupled CDNs in the presence of Hairpin a.** (a) Time-dependent fluorescence changes generated from the cleavage of the respective fluorophore/quencher-modified substrates by the DNAzyme reporter units associated with constituents AA′, BB′, AB′, BA′ included in the mixture of two CDNs X and Y upon subjecting the coupled CDNs to the trigger Hairpin a at different time intervals 0, 3, 6, 9, 12, 18, and 24 hours. (b) Time-dependent fluorescence changes generated from the cleavage of the respective fluorophore/quencher-modified substrates by the DNAzyme reporter units associated with constituents CC′, DD′, CD′, DC′ included in the mixture of two CDNs X and Y before (black lines) and after (red lines) the addition of Hairpin a to equilibrate the system for 24 hours.

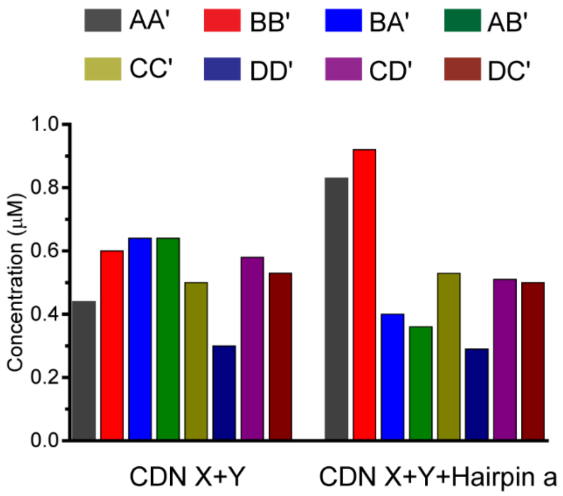

Supplementary Figure 18. **The concentrations of the constituents associated with the two CDNs X and Y (in the form of a bar presentation) before and after subjecting the coupled networks to Hairpin a after 24 hours.** By following the time-dependent fluorescence changes upon cleavage of the fluorophore/quencher (Fi/Qi)-functionalized substrates corresponding to the respective DNAzyme reporter units, and using appropriate calibration curves of the individual constituents, the quantitative evaluation of the concentrations of the constituents in the CDNs is accomplished.

Note, however, that under these conditions the cleavage of H_a by CC′ has no immediate effect on the performance of the metabolic module of CDN Y.

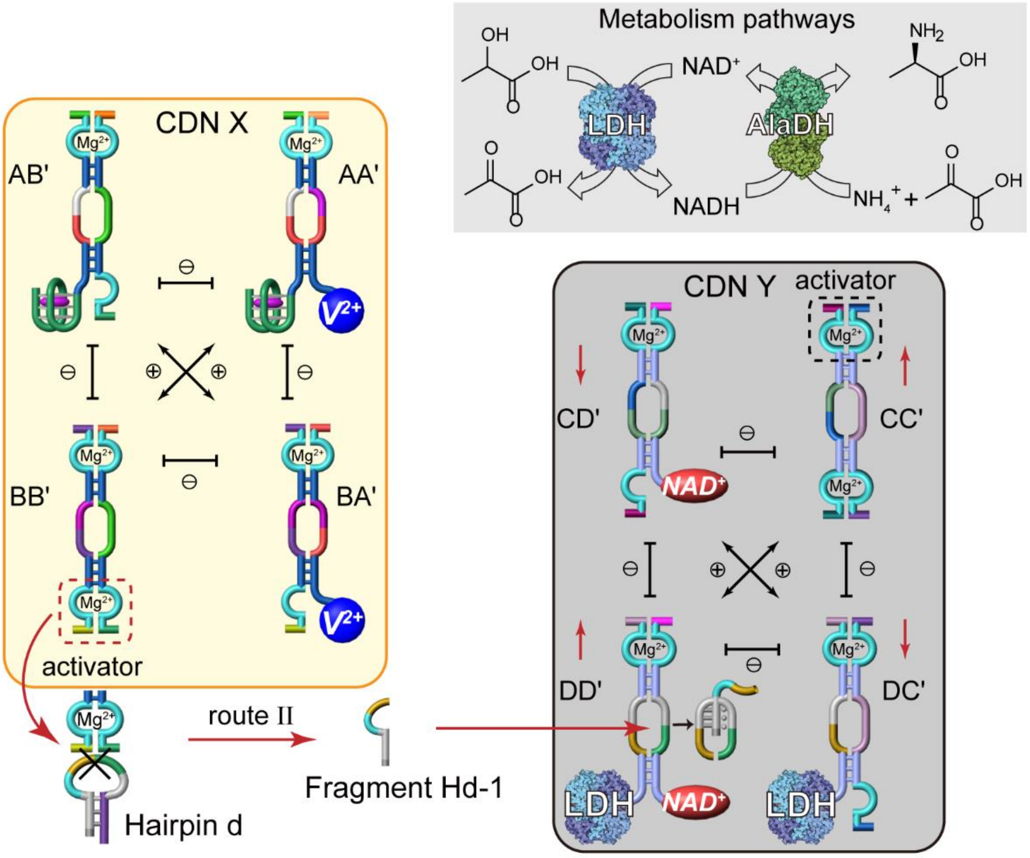

Supplementary Figure 19. **Schematic composition of intercommunicated control between two CDNs using hairpin d (H_d) as a trigger and control over the lactate/LDH/NAD$^+$ metabolic process.** Trigger hairpin d acts as a substrate for the DNAzyme activator associated with BB′ of CDN X. Trigger H_d acts as a substrate for the DNAzyme activator associated with BB′ of CDN X to yield the fragment strand H_{d-1} that interacts with DD′, resulting in the up-regulation of DD′ and enhanced activity of CDN Y.

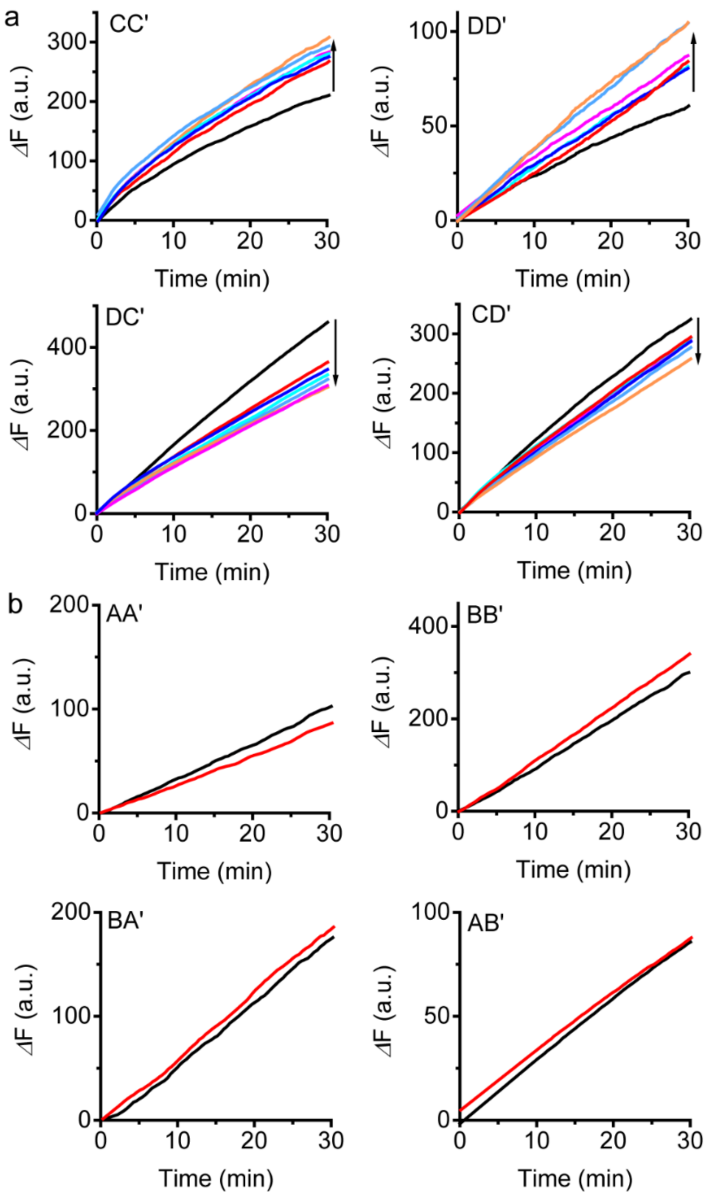

Supplementary Figure 20. **Time-dependent fluorescence changes generated from the cleavage of the respective fluorophore/quencher-modified substrates by the DNAzyme reporter units in the coupled CDNs in the presence of Hairpin d**. (a) Time-dependent fluorescence changes generated from the cleavage of the respective fluorophore/quencher-modified substrates by the DNAzyme reporter units associated with constituents CC′, DD′, CD′, DC′ included in the mixture of two CDNs X and Y upon subjecting the coupled CDNs to the trigger Hairpin d at different time intervals 0, 3, 6, 9, 12, 18, and 24 hours. (b) Time-dependent fluorescence changes generated from the cleavage of the respective fluorophore/quencher-modified substrates by the DNAzyme reporter units associated with constituents AA′, BB′, AB′, BA′ included in the mixture of two CDNs X and Y before (black lines) and after (red lines) the addition of Hairpin d to equilibrate the system for 24 hours.

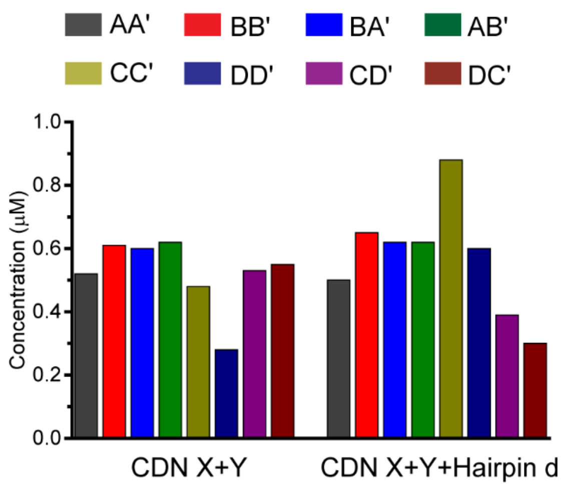

Supplementary Figure 21. **The concentrations of the constituents associated with the two CDNs X and Y (in the form of a bar presentation) before and after subjecting the coupled networks to Hairpin d after 24 hours.** By following the time-dependent fluorescence changes upon cleavage of the fluorophore/quencher (Fi/Qi)-functionalized substrates corresponding to the respective DNAzyme reporter units, and using appropriate calibration curves of the individual constituents, the quantitative evaluation of the concentrations of the constituents in the CDNs is accomplished.

Note, however, that the cleavage of H_d by the photosynthetic module has no immediate effect on the activity of CDN X.

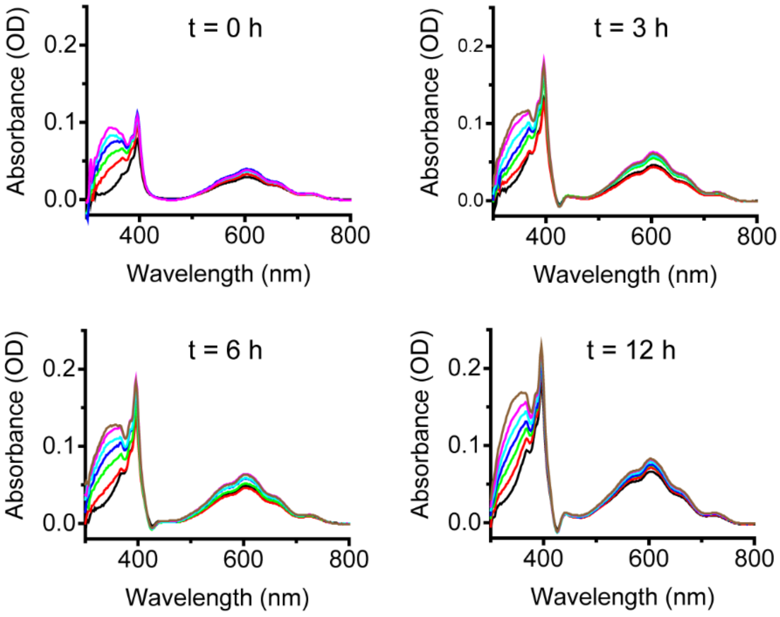

Supplementary Figure 22. **The biocatalytic transformation of NADPH in the coupled CDNs X and Y, in the presence of Ha.** Time-dependent absorption spectra corresponding to the photosensitized/FNR-synthesized NADPH by CDN X states generated at different time-intervals (0, 3, 6, 12 h) of exposure to CDN Y producing the H_{a-1}.

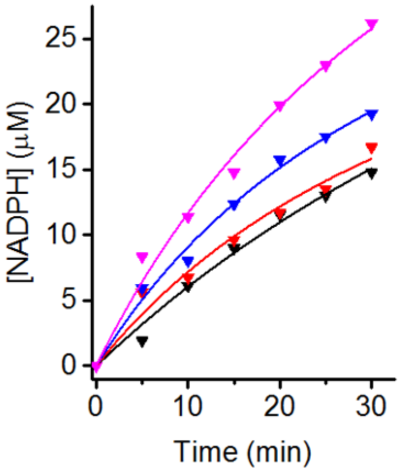

Supplementary Figure 23. **Duplicate results of experiments shown in Figure 3b.** Time-dependent formation of NADPH by the photosynthetic network at time-intervals of treatment of mixture M with the stimulant H_a.

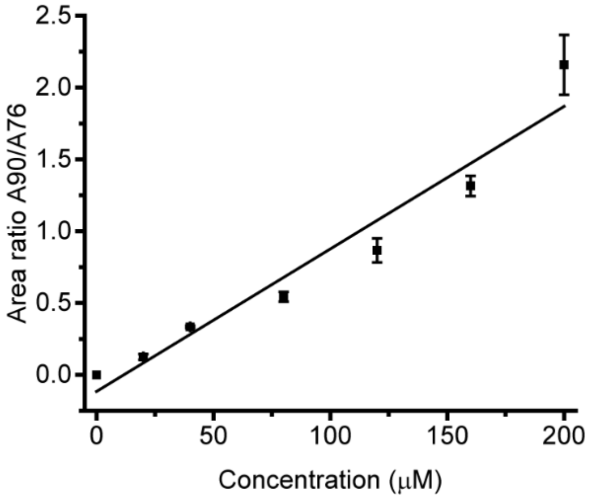

Supplementary Figure 24. **Calibration curve of the relations between the integrated area ratio A90/A76 (m/z = 90 (L-alanine) and m/z = 76 (glycine)) and different concentrations of L-alanine**. The calibration curve is used for the calculation of the concentration of L-alanine generated by coupled CDNs X and Y. Error bars, mean ± s.d. based on three independent experiments.

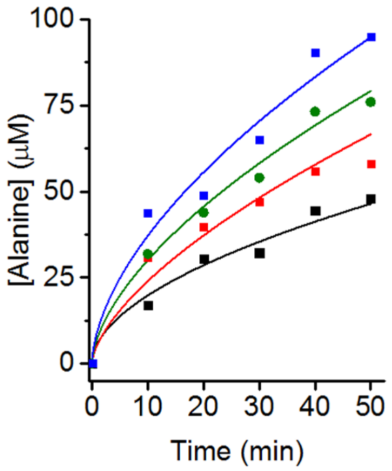

Supplementary Figure 25. **Duplicate results of experiments shown in Figure 3d**. Time-dependent formation of L-alanine by CDN Y at time-intervals of treatment of mixture M with the stimulant H_d.

Supplementary Table 6. **Mass analysis of L-alanine generated by coupled CDN mixtures before addition of Hairpin d.** A90 is the integrated area of mass peak at m/z = 90 (L-alanine), A76 is the integrated area of mass peak at m/z = 76 (glycine as an internal standard, positive mode).

Time (min)	CDN X+Y		
	A90	A76	A90/A76
10	21634	226634	0.095
	23414	462520	0.05
20	7357	38867	0.189
	31177	167011	0.1866
30	23211	120921	0.2
	27231	133019	0.205
40	17665	62805	0.28
	25504	78395	0.325
50	44373	120006	0.37
	40361	112459	0.36

Supplementary Table 7. Mass analysis of L-alanine generated by coupled CDN mixtures after addition of Hairpin d for 3 hours. A90 is the integrated area of mass peak at m/z = 90 (L-alanine), A76 is the integrated area of mass peak at m/z = 76 (glycine as an internal standard, positive mode).

Time (min)	CDN X+Y + Hairpin d (3h)		
	A90	A76	A90/A76
10	5684	41573	0.137
	30878	161028	0.192
20	30276	132132	0.229
	21345	76294	0.28
30	24228	64287	0.377
	27481	79269	0.35
40	44168	97999	0.45
	44201	91291	0.44
50	7886	13007	0.55
	9000	20916	0.43

Supplementary Table 8. **Mass analysis of L-alanine generated by coupled CDN mixtures after addition of Hairpin d for 6 hours.** A90 is the integrated area of mass peak at m/z = 90 (L-alanine), A76 is the integrated area of mass peak at m/z = 76 (glycine as an internal standard, positive mode).

Time (min)	CDN X+Y + Hairpin d (6h)		
	A90	A76	A90/A76
10	24280	130637	0.186
	5463	26991	0.2
20	18260	58888	0.31
	15464	48159	0.32
30	19544	44836	0.436
	35760	85163	0.42
40	66625	105883	0.6292
	8398	13772	0.61
50	33346	49426	0.67
	7302	11385	0.64

Supplementary Table 9. Mass analysis of L-alanine generated by coupled CDN mixtures after addition of Hairpin d for 12 hours. A90 is the integrated area of mass peak at m/z = 90 (L-alanine), A76 is the integrated area of mass peak at m/z = 76 (glycine as an internal standard, positive mode).

Time (min)	CDN X+Y + Hairpin d (12h)		
	A90	A76	A90/A76
10	10000	37572	0.27
	43035	133535	0.32
20	21749	55949	0.39
	54901	150468	0.365
30	20249	33518	0.6
	8452	15908	0.53
40	51305	67742	0.753
	52534	67457	0.779
50	82899	105470	0.786
	50396	62293	0.81

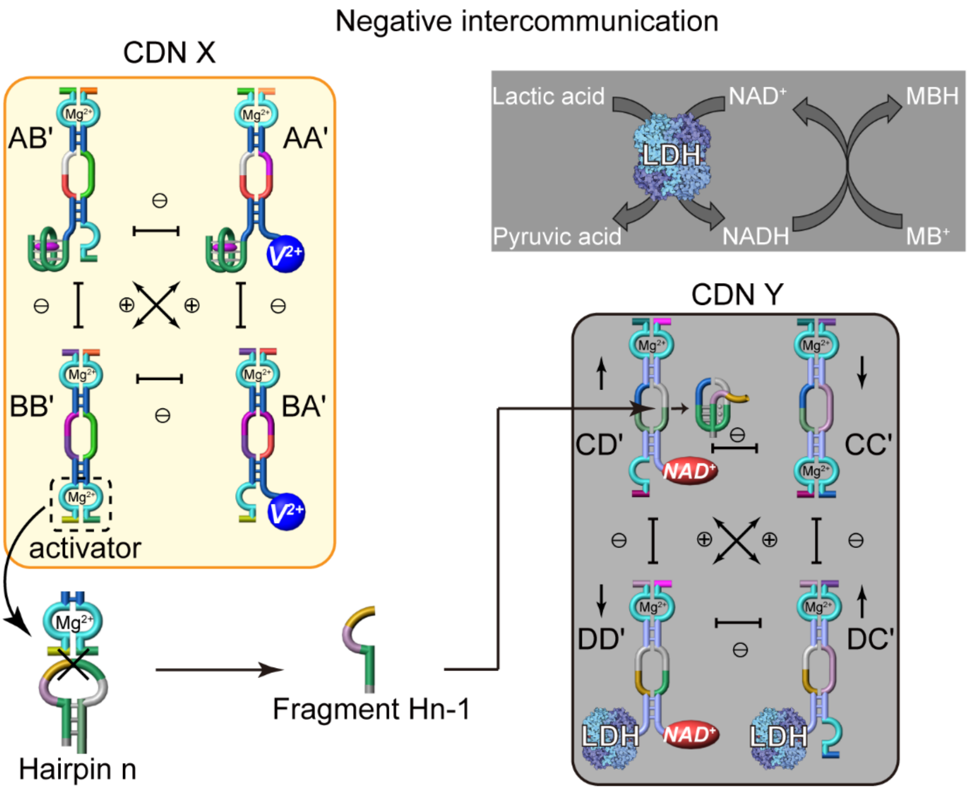

Supplementary Figure 26. Schematic composition of negative intercommunication between two CDNs using hairpin H_n as a trigger and control over the LDH/NAD^+/MB^+ cascade. Trigger hairpin H_n acts as a substrate for the DNAzyme activator associated with BB′ of CDN X to yield the fragment strand H_{n-1} that interacts with CD′, resulting in the up-regulation of CD′ and down-regulation of DD′.

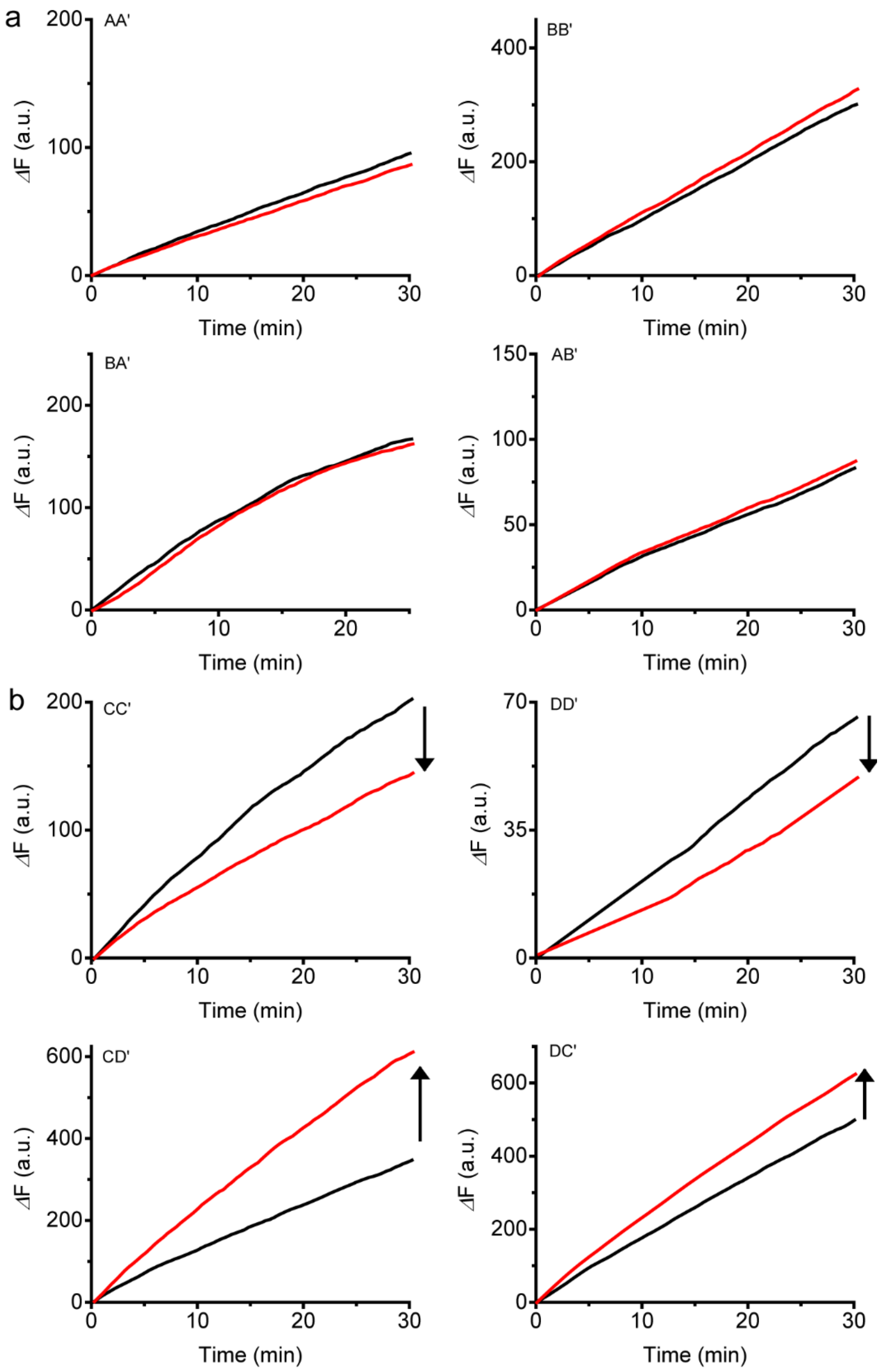

Supplementary Figure 27. **Time-dependent fluorescence changes generated from the cleavage of the respective fluorophore/quencher-modified substrates by the DNAzyme reporter units in the coupled CDNs in the presence of Hairpin n**. (a-b) Time-dependent fluorescence changes generated from the cleavage of the respective fluorophore/quencher-modified substrates by the DNAzyme reporter units associated with all constituents included in the mixture of two CDNs X and Y before (black lines) and after (red lines) the addition of Hairpin n to equilibrate the system for 24 hours.

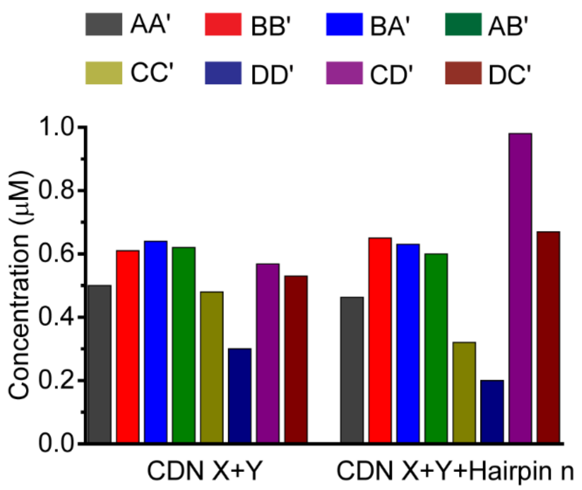

Supplementary Figure 28. **The concentrations of the constituents associated with the two CDNs X and Y (in the form of a bar presentation) before and after subjecting the coupled networks to Hairpin n after 24 hours.** By following the time-dependent fluorescence changes upon cleavage of the fluorophore/quencher (Fi/Qi)-functionalized substrates corresponding to the respective DNAzyme reporter units, and using appropriate calibration curves of the individual constituents, the quantitative evaluation of the concentrations of the constituents in the CDNs is accomplished.

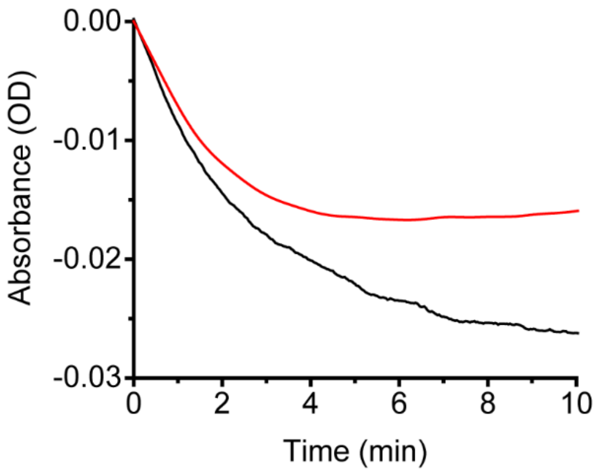

Supplementary Figure 29. **The biocatalytic reduction of MB^+ to MBH in the coupled CDNs X and Y in the presence of Hairpin n**. Time-dependent absorbance changes generated by the LDH/NAD^+/MB^+ cascade in the coupled CDNs X and Y before (black curve) and after (red curve) addition of trigger Hairpin n for 24 hours.

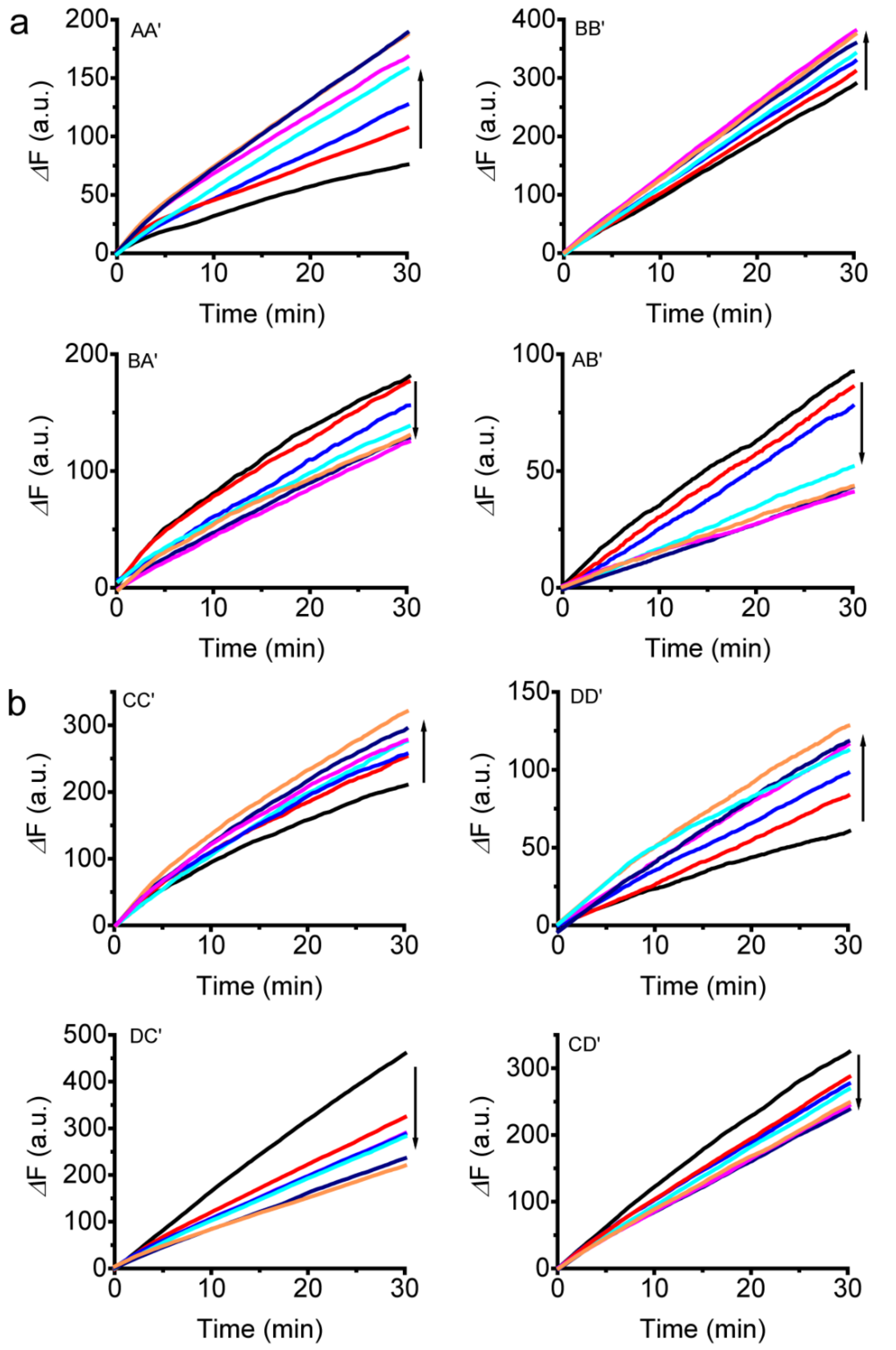

Supplementary Figure 30. **Time-dependent fluorescence changes generated from the cleavage of the respective fluorophore/quencher-modified substrates by the DNAzyme reporter units, in the presence of the triggers H_a and H_d.** (a-b) Time-dependent fluorescence changes generated from the cleavage of the respective fluorophore/quencher-modified substrates by the DNAzyme reporter units associated with all the constituents included in the mixture of two CDNs X and Y upon subjecting the coupled CDNs to the triggers H_a and H_d at different time intervals 0, 3, 6, 9, 12, 18, and 24 hours.

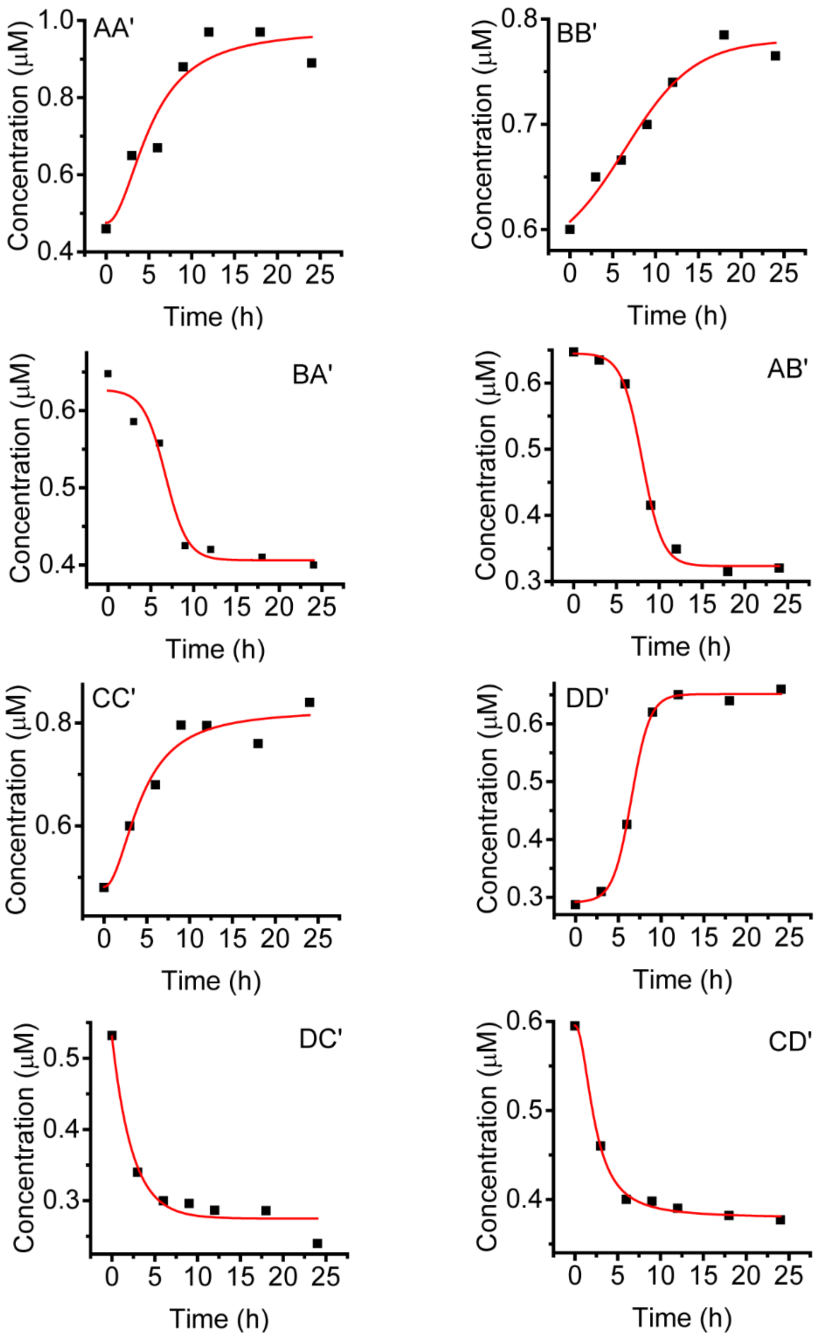

Supplementary Figure 31. **Time-dependent concentration changes of all the constituents in the coupled CDNs X and Y, in the presence of the triggers H_a and H_d.** Time-dependent concentration changes of each constituent in the mixture of two CDNs X and Y upon subjecting the coupled CDNs to the triggers H_a and H_d at different time intervals 0, 3, 6, 9, 12, 18, and 24 hours.

Supplementary Note

Supplementary Figure 31 shows the time-dependent concentrations of the constituents in CDNs X and Y upon subjecting the mixture of networks to the two hairpins H_a and H_d. Within a time-interval of 24 h, the concentrations of AA′, BB′ in CDN X and CC′, DD′ in CDN Y increase, while the concentrations of BA′, AB′, DC′ and CD′ decrease. After a time-interval of *ca.* 10 h, the concentrations of the constituents reach saturation. These results are consistent with the dynamic feedback-driven intercommunication of the two networks, where the cleavage of H_a leads to the up-regulation of AA′ and BB′, and the cleavage of H_d results in the up-regulation of DD′ and CC′. The saturation of the dynamic intercommunication of the CDNs presumably originates from the consumption of the hairpins.

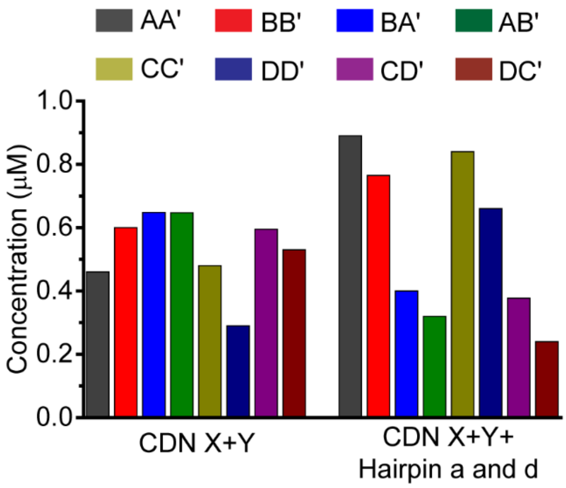

Supplementary Figure 32. **The concentrations of the constituents associated with the two CDNs X and Y (in the form of a bar presentation) before and after subjecting the coupled networks to Hairpin a and Hairpin d after 24 hours.** By following the time-dependent fluorescence changes upon cleavage of the fluorophore/quencher (Fi/Qi)-functionalized substrates corresponding to the respective DNAzyme reporter units, and using appropriate calibration curves of the individual constituents, the quantitative evaluation of the concentrations of the constituents in the CDNs is accomplished.

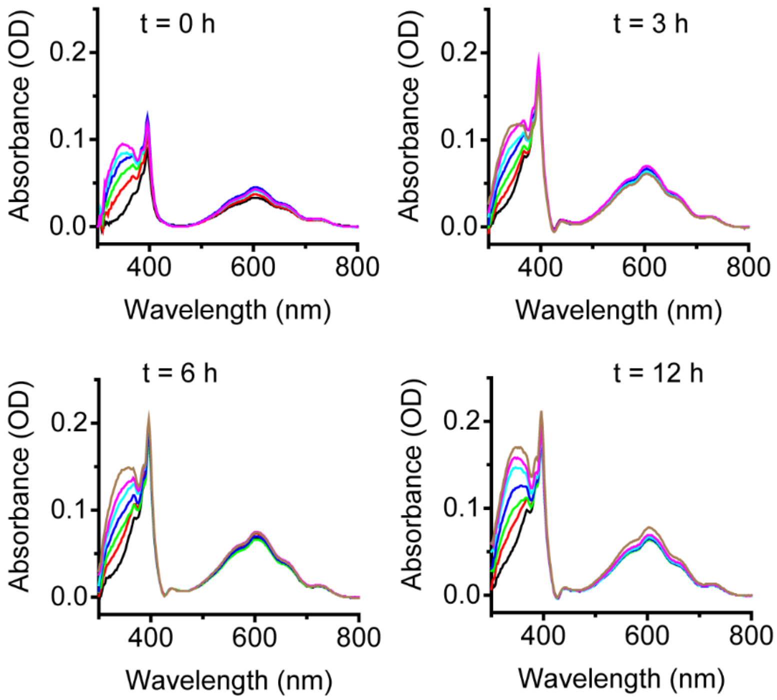

Supplementary Figure 33. **The biocatalytic transformation of NADPH in the coupled CDNs X and Y, in the presence of Ha and Hd.** Time-dependent absorbance spectra of NADPH generated by the photosynthetic module at time-intervals (0, 3, 6, 12 h) of the feedback-driven intercommunication of the two networks.

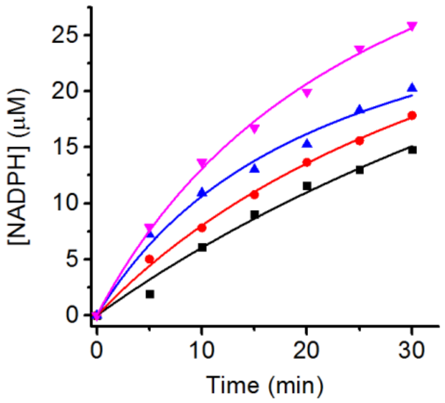

Supplementary Figure 34. **Duplicate results of experiments shown in Figure 4b**. Time-dependent formation of NADPH by the photosynthetic network X at time-intervals of activation by stimulant H_a-triggered operation of the metabolic network Y.

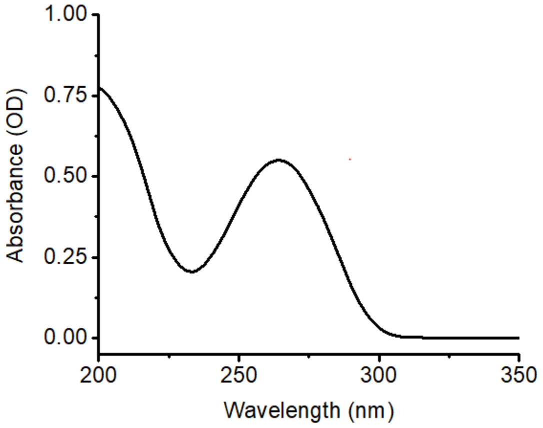

Supplementary Figure 35. UV-vis spectrum of the modified strand A′-V^{2+}. The absorbance at 260 and 280 nm are used for the quantification of the DNA labeling ratio of the purified V^{2+}-DNA conjugates.

Supplementary Table 10. Quantification of the DNA labeling ratio of the purified V^{2+}-DNA conjugates by measuring the absorbance at 260 and 280 nm shown in Supplementary Figure 35

	ε_{260} (M^{-1}/cm)	ε_{280} (M^{-1}/cm)		ε_{260} (M^{-1}/cm)	ε_{280} (M^{-1}/cm)	A260	A280	Average coupling ratio (DNA: V^{2+})
V^{2+}	20,700	13,600	A′	513,300	318,470	0.535	0.333	1.1

$$A_{260}\ (V^{2+}\text{-DNA}) = \varepsilon_{260}\ (V^{2+}) \times C\ (V^{2+}) + \varepsilon_{260}\ (\text{DNA}) \times C\ (\text{DNA})$$

$$A_{280}\ (V^{2+}\text{-DNA}) = \varepsilon_{280}\ (V^{2+}) \times C\ (V^{2+}) + \varepsilon_{280}\ (\text{DNA}) \times C\ (\text{DNA})$$

$$\text{Ratio}\ \left(\frac{DNA}{\text{V2+}}\right) = \frac{C\ (DNA)}{c\ (\text{V2+})}$$

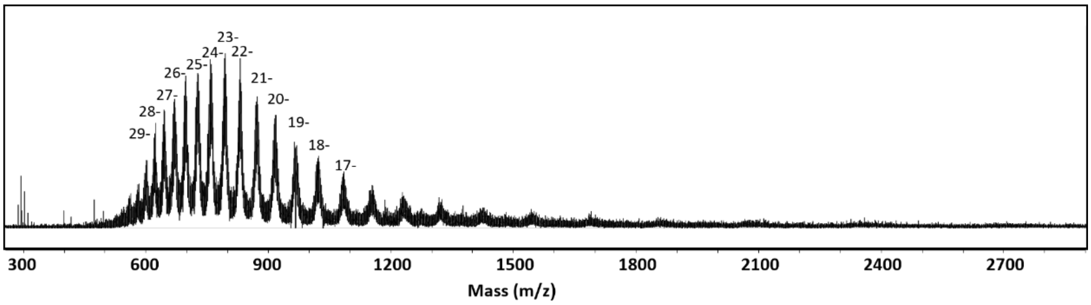

Supplementary Figure 36. Mass analysis of A′-V^{2+}. The sample shows the peaks that carrying negative charges.

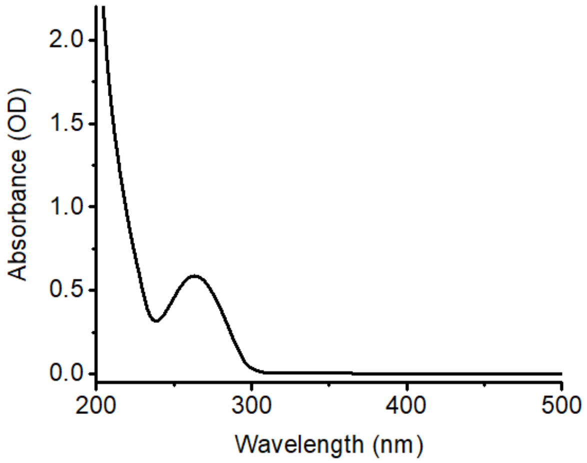

Supplementary Figure 37. **UV-vis spectrum of the modified strand D-LDH**. The absorbance at 260 and 280 nm are used for the quantification of the DNA labeling ratio of the purified enzyme-DNA conjugates.

Supplementary Table 11. Quantification of the DNA labeling ratio of the purified enzyme-DNA conjugates by measuring the absorbance at 260 and 280 nm shown in Supplementary Figure 37

	ε_{260} (M^{-1}/cm)	ε_{280} (M^{-1}/cm)		ε_{260} (M^{-1}/cm)	ε_{280} (M^{-1}/cm)	A260	A280	Average coupling ratio (DNA: LDH)
LDH	132,175	186,502	D	500,300	302,615	0.57	0.423	1.28

A_{260} (Enzyme-DNA) = ε_{260} (enzyme) × C (enzyme) + ε_{260} (DNA) × C (DNA)

A_{280} (Enzyme-DNA) = ε_{280} (enzyme) × C (enzyme) + ε_{280} (DNA) × C (DNA)

$$\text{Ratio} \left(\frac{DNA}{enzyme}\right) = \frac{C\ (DNA)}{c\ (enzyme)}$$

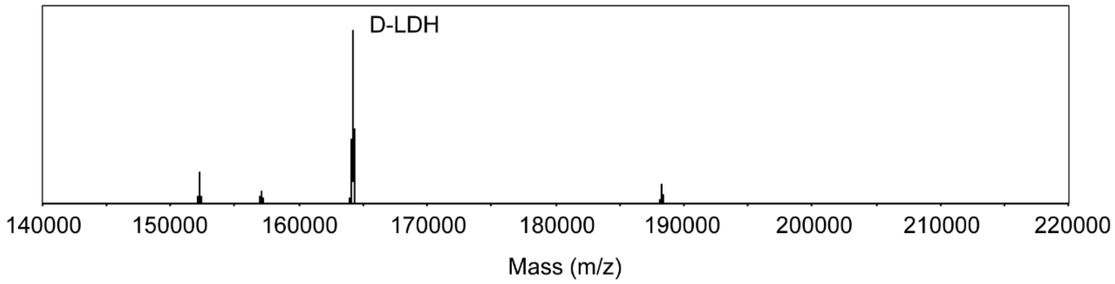

Supplementary Figure 38. **Mass analysis of D-LDH.** The main peak shows the product of D-LDH.

Supplementary Table 12. Strands of CDNs X and Y *(5′→3′)*

A	GATATCAGCGATCAGTAAACACTTTATTTAA*TTTCTCCTTT*CCACAAATGACTTTTGGGTTGGGCGGGATGGG
A′	SH-TTTGTCATTTGTAA*TTTCCTCTTTTCTTCCTT*AATGTTTACTGCACCCATGTTACTCT
B	CTGCTCAGCGATCAGTAAACACC*TTCCTTCTTT* TATTTATTTAACAAATGACCACCCATGTTCTGTC
B′	CAACTCAGCGATGTCATTTGTATTTATTTATTTATTTATTTAATGTTTACTGCACCCATGTTCGTCA
C	GTCCTCAGCGATCTCAAATTGACTTATTATCTTTCCTTTTCCGTTAAACACCACCCATGTTGAGTG
C′	GTAGTCAGCGATGTGTTTAACCTTTATTTATTTATTTATTTCCAATTTGAGCACCCATGTTTCAGT
D	CTGTTCAGCGATCTCAAATTGACT*TCTCTTCTTT*ATTTATTAGTTAAACACTTT-SH
D′	SH-TTTGTGTTTAACCATTTTCC*TTTCTTCTCT*TACCAATTTGAGCACCCATGTTCCTGA

The respective Mg^{2+}-dependent DNAzyme bases are underlined and the T-A·T triplex domains associated with AA′, BA′, DD′ and CD′ are presented in italic. G-quadruplex bases in strand A are marked as pink.

Supplementary Table 13. Strands of triggers *(5′→3′)*

T_1	AGCAAAGGAGAAACATACACTCAT
T_1'	ATGAGTGTATGTTTCTCCTTTGCT
T_2	TGCAAGGAAGAAACATACACTCATAGG
T_2'	CCTATGAGTGTATGTTTCTTCCTT
T_3	ATGCAAAGAAGAGACATAGACAGATAGACAG
T_3'	CTGTCTATCTGTCTATGTCTCTTCTTT
T_4	TGCAAAAGGAAAGCATAGACAGATAGG
T_4'	CCTATCTGTCTATGCTTTCCTTTTGCA

Supplementary Table 14. Strands of hairpins *(5′→3′)*

H_a	AGCAAAGGAGAAACATACACTCAT**rA**GGACTACAAACAATCTCCTTTGCTAT
H_d	ATGCAAAGAAGAGACATAGACAGAT**rA**GGAGTTGAAACAATCTTCTTTGCATA
H_n	ATGCAAAAGGAAAGCATAGACAGAT**rA**GGAGTTGAAACAATTCCTTTTGCATA

Supplementary Table 15. Strands of substrates for Mg^{2+}-dependent DNAzyme reporter units *(5′→3′)*

sub1 (AA′)	FAM-AGAGTAT**rA**GGATATC-BHQ1
sub2 (BB′)	ROX-TGACGAT**rA**GGAGCAG-BHQ2
sub3 (BA′)	Cy5-AGAGTAT**rA**GGAGCAG-BHQ2
sub4 (AB′)	Cy5.5-TGACGAT**rA**GGATATC-IBRQ
sub5 (DC′)	FAM-ACTGAAT**rA**GGAACAG-BHQ1
sub6 (CD′)	ROX-TCAGGAT**rA**GGAGGAC-BHQ2
sub7 (CC′)	Cy5-ACTGAAT**rA**GGAGGAC-BHQ2
sub8 (DD′)	Cy5.5-TCAGGAT**rA**GGAACAG-IBRQ

The ribonucleobase cleavage site, **rA**, in the substrates of the different Mg^{2+}-dependent DNAzymes is indicated in bold.

Supplementary References

[1] L. A. Kelly, M. A. J. Rodgers. *J. Phys. Chem*. 1994,98, 6386-6391.

[2] C. Wang, L. Yue, I. Willner. *Nat. Catal*. 2020, 3, 941-950.
